# Species-specific roles for the MAFA and MAFB transcription factors in regulating islet **β** cell identity

**DOI:** 10.1172/jci.insight.166386

**Published:** 2023-08-22

**Authors:** Jeeyeon Cha, Xin Tong, Emily M. Walker, Tehila Dahan, Veronica A. Cochrane, Sudipta Ashe, Ronan Russell, Anna B. Osipovich, Alex M. Mawla, Min Guo, Jin-hua Liu, Zachary A. Loyd, Mark O. Huising, Mark A. Magnuson, Matthias Hebrok, Yuval Dor, Roland Stein

**Affiliations:** 1 Division of Diabetes, Endocrinology, and Metabolism, Vanderbilt University Medical Center, Nashville, Tennessee, USA.; 2 Department of Molecular Physiology and Biophysics, Vanderbilt University, Nashville, Tennessee, USA.; 3 Department of Developmental Biology and Cancer Research, The Hebrew University-Hadassah Medical School, Jerusalem, Israel.; 4 Diabetes Center, Department of Medicine, University of California, San Francisco, San Francisco, California, USA.; 5 Department of Neurobiology, Physiology & Behavior, College of Biological Sciences, University of California, Davis, Davis, California, USA.

**Keywords:** Cell Biology, Endocrinology, Beta cells, Diabetes, Islet cells

## Abstract

Type 2 diabetes (T2D) is associated with compromised identity of insulin-producing pancreatic islet β cells, characterized by inappropriate production of other islet cell–enriched hormones. Here, we examined how hormone misexpression was influenced by the MAFA and MAFB transcription factors, closely related proteins that maintain islet cell function. Mice specifically lacking MafA in β cells demonstrated broad, population-wide changes in hormone gene expression with an overall gene signature closely resembling islet gastrin^+^ (Gast^+^) cells generated under conditions of chronic hyperglycemia and obesity. A human β cell line deficient in MAFB, but not one lacking MAFA, also produced a GAST^+^ gene expression pattern. In addition, GAST was detected in human T2D β cells with low levels of MAFB. Moreover, evidence is provided that human MAFB can directly repress *GAST* gene transcription. These results support a potentially novel, species-specific role for MafA and MAFB in maintaining adult mouse and human β cell identity, respectively. Here, we discuss the possibility that induction of Gast/GAST and other non–β cell hormones, by reduction in the levels of these transcription factors, represents a dysfunctional β cell signature.

## Introduction

The pancreatic islet is composed of endocrine cells expressing distinct peptide hormones, such as insulin from β cells, glucagon from α cells, and somatostatin (SST) from δ cells, which play critical roles in maintaining euglycemia. Experiments conducted principally in mouse models have demonstrated that combinatorial expression of islet-enriched transcription factors (TFs), such as Pdx1, Nkx6.1, Arx, Hhex, and MafA, are essential in the developmental and/or adult function of islet cell types (1, 2). For example, studies performed on *MafA* mutant mice (global *MafA*^–/–^, pancreas-specific *MafA^∆panc^*, and β cell–specific *MafA*^∆β^) established a key role for this TF in activating programs promoting β cell maturation and glucose-stimulated insulin secretion (GSIS) (1, 3). In contrast, while the related MafB protein is present throughout the life span of the islet α cell, this TF is only produced in murine β cells embryonically, except when transiently expressed during pregnancy to facilitate β cell expansion (4, 5). Moreover, MafB cannot rescue MafA function in MafA-deficient β cells (4), likely due to unique coregulator interactions within their distinct C-terminal region sequences (6).

While expression of most islet-enriched TFs is similar between rodents and humans, the MAFA and MAFB proteins have different temporal profiles in humans. Consequently, MAFB is expressed throughout the life span of both human α and β cells, and this contrasts from the postnatal rodent MafA^+^MafB^–^ β cell population (1). In addition, MAFA protein is not readily detectable in human islet β cells until approximately 9 years of age (4), while in rodents, this TF is expressed at the onset of insulin^+^ cell formation during embryogenesis (7). In contrast to rodents, human MAFB appears to be essential for β cell development, as supported by the inability of human embryonic stem cells (hESCs) lacking MAFB to produce insulin (INS^+^) cells despite being subject to a well-established β cell differentiation protocol (8). Strikingly, these mutant cells still produce many islet-enriched TFs associated with *INS* transcription (e.g., NKX6.1 and PDX1). In addition, a subpopulation of MAFB^KO^ β-like cells express non–β cell hormones, such as SST, pancreatic polypeptide (PP, encoded by *PPY*), and GAST, with the latter principally expressed in adult gastric (G) cells and only transiently expressed in the pancreas during islet formation embryonically (9).

Since synthesis of non–β cell hormones appears to be negatively regulated by human MAFB (8) and is associated with the loss of β cell identity and activity postnatally in type 2 diabetes (T2D) (10–14), we examined here how MAFA and/or MAFB contributed to this process in adult mice and human islets. Non–β cell hormone–producing cells were not only inappropriately generated in mouse *MafA*^∆β^ islets but were also detected under pathophysiologic conditions of hyperglycemia and obesity that suppress expression of this TF. Notably, these conditions of stress also induced the production of gastrin^+^ (Gast^+^) cells in mouse islets, and these cells shared a gene expression profile with the broader *MafA*^∆β^ β cell population, even though only a small fraction of *MafA*^∆β^ islet cells were Gast^+^. *SST*, *PPY*, and *GAST* were also inappropriately expressed upon knockdown of MAFB, but not MAFA, in the human EndoC-βH2 β cell line. Furthermore, both SST^+^ and GAST^+^ cells were observed in β cells in human T2D islets. Because MAFA and MAFB protein levels are more sensitive to metabolic and oxidative stress than other islet-enriched TFs (15), we discuss the possibility that their loss under such conditions contributes to changes in β cell identity and inactivity associated with T2D progression.

## Results

### Non–β cell hormone expression is induced in mouse MafA^∆β^ islets.

Our earlier results had shown that differentiation of the male human embryonic MEL-1 stem cell line to β-like cells requires MAFB, with MAFB-deficient β-like cells having unanticipated features, including loss of INS production and generation of cells producing several non–β cell hormones (8). Since MafA, but not MafB, is present in the postnatal mouse β cell, we sought to determine if MafA had similar regulatory properties in postnatal mouse islet β cells. A β cell–specific deletion mutant was generated by crossing *MafA^fl/fl^* mice (3) with *Ins1^Cre^* mice (16), effectively removing this TF from male and female islet β cells (termed *MafA*^∆β^) (Supplemental Figure 1, A and B; supplemental material available online with this article; https://doi.org/10.1172/jci.insight.166386DS1). Notably, this *Ins1^Cre^* line allows deletion only in the pancreatic β cell lineage, unlike other *Insulin* enhancer/promoter–driven Cre recombinase mouse lines that misexpress the transgene to drive recombination in the brain (17). As reported earlier for other targeted β cell *MafA*^∆β^–deficient mice (3, 4, 7, 18, 19), both male and female *MafA*^∆β^ mice manifested impaired glucose tolerance without overt hyperglycemia (Supplemental Figure 1C).

**Figure 1:**
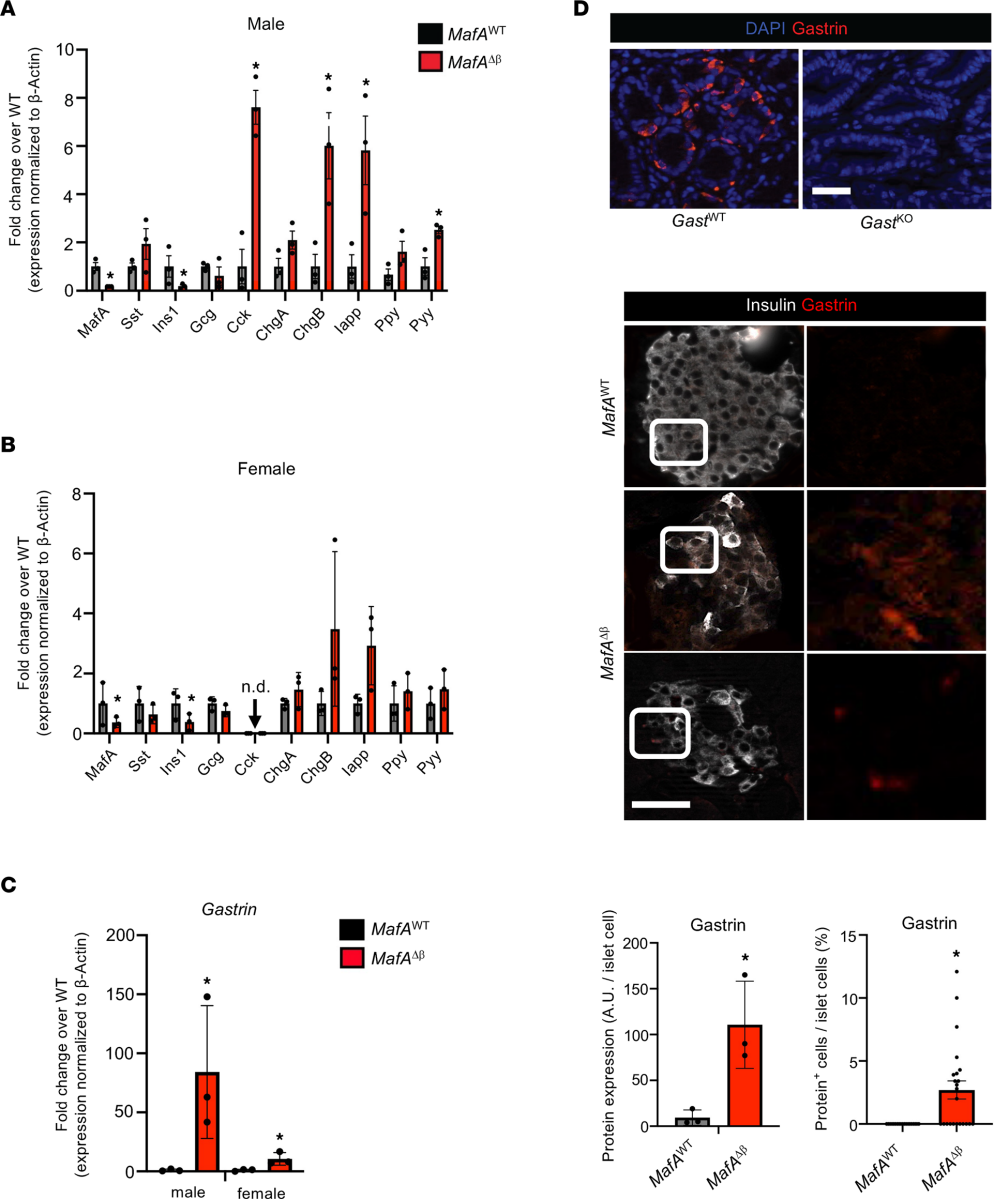
Non–β cell hormone expression is increased in *MafA*^∆β^ islets. (**A** and **B**) Analysis of *MafA*, *Insulin 1* (*Ins1*), and non–β cell hormone mRNA levels in *MafA*^WT^ and *MafA*^∆β^ islets from 3-month-old adult male (**A**) and female (**B**) mice. Data are shown as mean ± SEM. *n* = 3–4 animals/group. *P < 0.05 by Student’s *t* test; n.d., not detectable. (**C**) *Gast* mRNA was induced more in male than in female *MafA*^∆β^ islets. Data are shown as mean ± SEM. *n* = 3–4 animals/group. *P < 0.05 by Student’s *t* test. (**D**) Top panel: Stomach G cells from *GAST*^WT^ and *GAST*^KO^ mice served as positive and negative control for Gast antibody staining. *n* = 2–3 animals/group. Middle panels: Representative images for Gast (red) and Insulin (white) immunostaining in male *MafA*^WT^ and *MafA*^∆β^ islets. Insets for Gast staining are shown in right panels. Bottom left panel: Gastrin signal (A.U.) per mouse islet cell. Bottom right panel: Gast^+^ cells per islet (% of islet cells). Four to 6 islets per mouse. *n* = 3–4 animals/group were quantified using ImageJ. Scale bar, 50 μm. Data are shown as mean ± SEM. *P < 0.05 by Student’s *t* test.

Analysis of endocrine cell hormone mRNA expression in FACS-purified β cells from adult *MafA*^∆β^ islets revealed that *Ins* levels were reduced, while expression of non–β cell hormones such as *cholecystokinin* (*Cck*), *chromogranin B* (*Chgb*), *peptide YY* (*Pyy*), and *Gast* were elevated (Figure 1, A–C). Interestingly, a profound sexual bias was observed for *Cck* (Figure 1, A and B) and *Gast* (Figure 1C). Gast protein was also detectable in male and female *MafA*^∆β^ islets but not *MafA*^WT^ (Figure 1D). However, gene markers associated with β cell dedifferentiation, such as *Ngn3*, *FoxO1*, and *Aldh1a3* (20, 21), were not detectable in *MafA*^∆β^ mice of either sex (Supplemental Figure 2). Notably, Gast expression was not detected in various models of mouse β cell dedifferentiation, such as in the context of Aldh1a3^+^ cells (20) or a vitamin D–binding protein (DBP) in a model of multiparity-induced diabetes (22) mice. Collectively, these results suggest that MafA represses postnatal production of hormones that are normally only transiently expressed during pancreatic β cell development and predominately elsewhere in the adult, such as *Gast* (duodenum, stomach) (9) and *Cck* (small intestine) (23). These data further indicate that MafA deficiency not only compromises adult mouse β cell activity but also β cell identity rather than dedifferentiation.

### The gene signature of the Gast^+^ cells produced in male insulin-resistant mice is found within the MafA^∆β^ β cell population.

**Figure 2:**
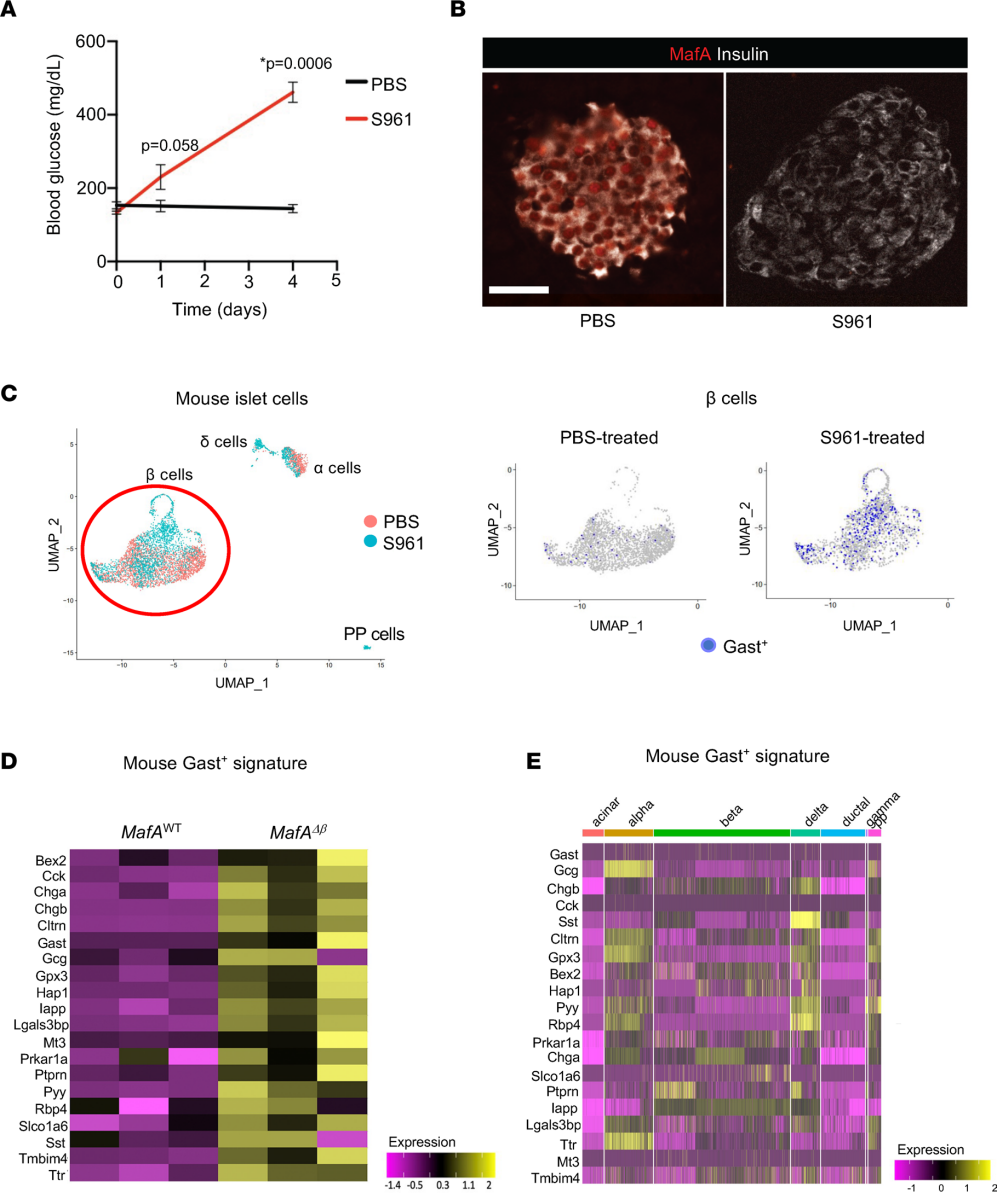
Islet Gast^+^ cells produced in insulin-resistant mice share molecular signatures with the broader *MafA*^∆β^ islet population. (**A**) Administration of the S961 insulin receptor antagonist for 4 days elevated random blood glucose levels in relation to PBS-treated control mice. Data are shown as mean ± SEM. *n* = 2–3 animals/group. *P < 0.05 by Student’s *t* test. (**B**) MafA (red) protein levels were markedly reduced in S961-treated mice. Insulin (white). *n* = 2–4 animals/group. Scale bar, 50 μm. (**C**) UMAP analyses of the single-cell RNA-Seq data in male S961-treated islets. Left panel: Cluster annotation shows that ~80% of islet cells were Insulin^+^ β cells (left, circled in red). Right panels: Gast^+^ cells (blue dots) were enriched in the β cell population. (**D**) Heatmap showing the 20 upregulated genes found in S961 Gast^+^ cells by single-cell RNA sequencing (Supplemental Table 1) were also elevated in male *MafA*^∆β^ islets. *MafA*^∆β^ β cell RNA-Seq were was used in this analysis. *n* = 3 animals/experimental group. (**E**) Heatmap showing limited overlap between the upregulated genes found in S961 Gast^+^ cells and other exocrine and islet cell types.

Administration of the insulin receptor antagonist S961 imparts hyperinsulinism and marked hyperglycemia in male mice (Figure 2A) (24–26) with a concomitant reduction of islet β cell MafA protein and mRNA levels (Figure 2B and data not shown). Four days of S961 treatment also stimulated the production of a rare Gast^+^ cell population (Figure 2C). Single-cell RNA-Seq revealed 20 genes significantly enriched in Gast^+^ cells compared with Gast^–^ β cells, including the *Gcg*, *Sst*, *Cck*, *Chga*, *Chgb*, *Pyy*, and *Iapp* endocrine markers (2,984 and 2,355 cells for PBS- and S961-treated cells, respectively; Supplemental Table 1). While female islets from S961-treated mice were not characterized here, the purified β cell population from male and female *ob/ob* mice — which represents a genetic model of obesity-induced glucose intolerance that manifests hyperglycemia, elevated plasma insulin (27), and loss of β cell MafA (28), akin to S961 treatment — were analyzed. The male, but not female, *ob/ob* β cell population expressed many of the genes present in male S961-induced Gast^+^ cells (Supplemental Figure 3A).

Like *ob/ob* islet β cells, the genes associated with S961-treated mouse islet Gast^+^ cells were also enriched in male *MafA*^∆β^ islet β cells sorted by FACS (Figure 2D). Importantly, while the S961-induced Gast^+^ gene signature included several genes found in other islet cell types (i.e., *Gcg*, *Sst*, *Cck*, *Chga*, *Chgb*, *Pyy*, and *Iapp*), there was little likeness at the gene expression level with naturally resident mouse islet α, δ, and PP cells (Figure 2E and Supplemental Table 1). Although Gast itself was not broadly produced in the male *MafA*^∆β^ islet β cell population (Figure 1C), our results indicate that these cells express genes associated with the Gast^+^ cells generated in hyperglycemic and obese mouse models (i.e., S961-treated and *ob/ob* mice). Unfortunately, mouse single-cell transcriptome data are currently unavailable for the Gast^+^ cells produced normally in the developing pancreas or more predominant stomach and duodenum G cells for comparison with the pathological gene signatures identified here in S961 and *ob/ob* islets. Of note, and as expected from analysis of hormone production in *MafA*^∆β^ islet β cells (Figure 1), female *MafA*^∆β^ β cells had less similarity at the gene expression level compared with these Gast^+^ cells than their male counterpart (Figure 2D and Supplemental Figure 3B).

### MAFB, and not MAFA, prevents GAST, SST, and PPY gene expression in human β cells.

Mouse β cells predominantly produce MafA in postnatal life, and mouse models deficient in pancreatic MafB are euglycemic and only display a significant islet phenotype during pregnancy when this TF is induced transiently (5, 6). In contrast, postnatal human islet β cells express both MAFA and MAFB, and human MAFA is not expressed until after the juvenile period (4). To provide insight into the role of these proteins in controlling non–β cell hormone expression in human β cells, MAFA and MAFB knockdown experiments were performed in the EndoC-βH1 (29) and EndoC-βH2 (30) β cell lines. Notably, EndoC-βH1 cells express both MAFA and MAFB and secrete insulin in response to high glucose or other secretagogues (29), while EndoC-βH2 cells only produce MAFB and are not glucose responsive until conditional Cre recombinase–mediated excision of their immortalizing transgenes hTERT and SV40 (30). Cre-activated EndoC-βH2 cells were not analyzed here.

**Figure 3:**
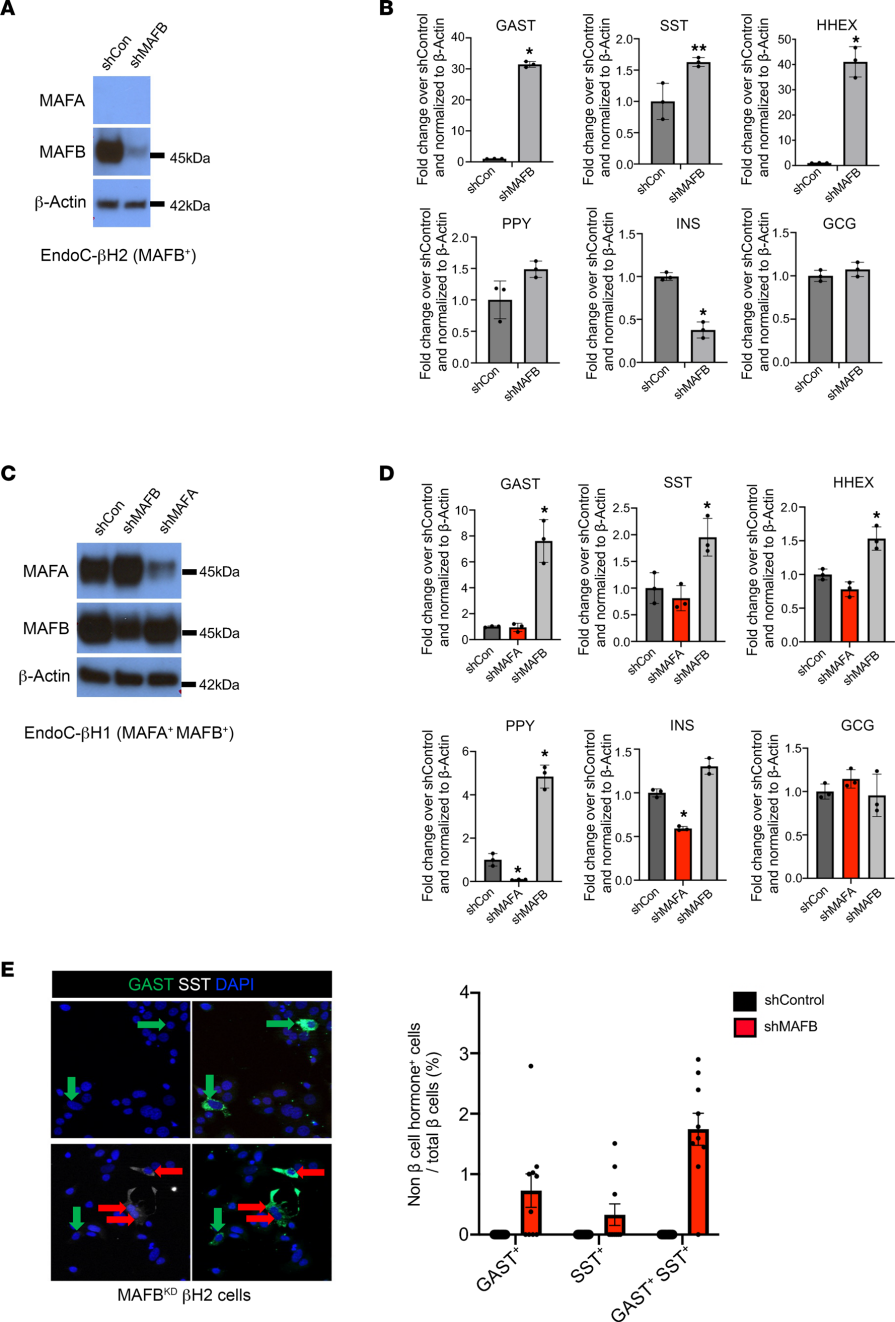
MAFB^KD^, but not MAFA^KD^, induces non–β cell hormone expression in human β cells. (**A**–**D**) Analysis of MAFA/B protein (**A** and **C**) and hormone-related mRNA levels (**B** and **D**) in shControl (scrambled construct), shMAFA-, or shMAFB-treated EndoC-βH2 (i.e., only MAFB^+^) (**A**) and EndoC-βH1 (i.e., MAFA^+^MAFB^+^) cells (**C**). β-Actin served as the internal control and expression was normalized to shControl levels. MAFA knockdown in shMAFA-treated cells was reduced by ~55% of the control level, and shMAFB-treated cells decreased by ~71%. Data are shown as mean ± SEM. *P < 0.05, **P < 0.005 by Student’s *t* test in **B**. *P < 0.05 by 2-way ANOVA in **D**. *n* = 2–3 replicates/experiment, and experiments were repeated 4 times. (**E**) Immunostaining for SST (white) and GAST (green) in MAFB^KD^ EndoC-βH2 cells. These proteins were undetectable in shControl-treated cells. Magnification, 20***×***. Left: Green arrows denote GAST^+^ SST^–^ cells, while red arrows denote GAST^+^ SST^+^ cells. Right: Quantification of hormone^+^ cells shown in MAFB^KD^ EndoC-βH2 cells relative to the total β cell number compared with shControl. Data are shown as mean ± SEM. *n* = 2–3 replicates/experiment, and experiments were repeated 4 times.

As predicted from the analysis of hESC-derived β-like cells lacking MAFB (MAFB^KO^) (8), lentivirus-mediated knockdown of MAFB protein (MAFB^KD^) in EndoC-βH2 β cells (Figure 3A) elevated *GAST*, *SST*, and *PPY* (increased trend) mRNA levels and decreased *INS* (Figure 3B, light gray bars). An analogous regulatory pattern was observed upon knockdown of MAFB in EndoC-βH1 cells (Figure 3, C and D, gray bars). GAST protein levels were increased in MAFB^KD^ EndoC-βH2 cells compared with control cells (Supplemental Figure 4). Notably, immunostaining results showed that only a small fraction of MAFB^KD^ EndoC-βH2 cells express GAST (~2%) or SST (~2%) (Figure 3E). GAST and SST (GAST^+^SST^+^) copositive cells were also observed in this setting (Figure 3E), similar to T2D islets (31). As expected, neither GAST nor SST protein was detected in shControl-treated EndoC-βH2 cells (Figure 3E).

In contrast to MAFB depletion, EndoC-βH1 cells deficient in MAFA did not generate a significant change in *GAST* or *SST* mRNA levels (Figure 3D, red bars), even though MAFA protein levels were effectively reduced (Figure 3C). However, the reduction in MAFA did decrease *INS* and *PYY* mRNA production in MAFA^KD^ EndoC-βH1 cells. Our data suggest that β cell MAF TFs prevent non–β cell hormone gene expression in a species-specific manner, with only MafA serving in this capacity in mice and MAFB principally in humans.

Because MAFA and MAFB recruit the mixed-lineage leukemia 3 (MLL3) and MLL4 methyltransferase coregulator complexes to alter chromatin structure and gene expression (6), we analyzed whether *GAST* expression was regulated by this complex in MAFB^KD^ EndoC-βH2 cells. Knockdown of a core subunit of these complexes, NCOA6, did not influence MAFB^KD^-regulated *GAST* expression (Supplemental Figure 5). Future studies to identify the coregulators mediating the repressive effects of MafA and MAFB in β cells are warranted (6).

### MAFB^KD^ EndoC-βH2 cells produce a gene signature reminiscent of the GAST^+^ MAFB^KO^ β-like cells.

**Figure 4:**
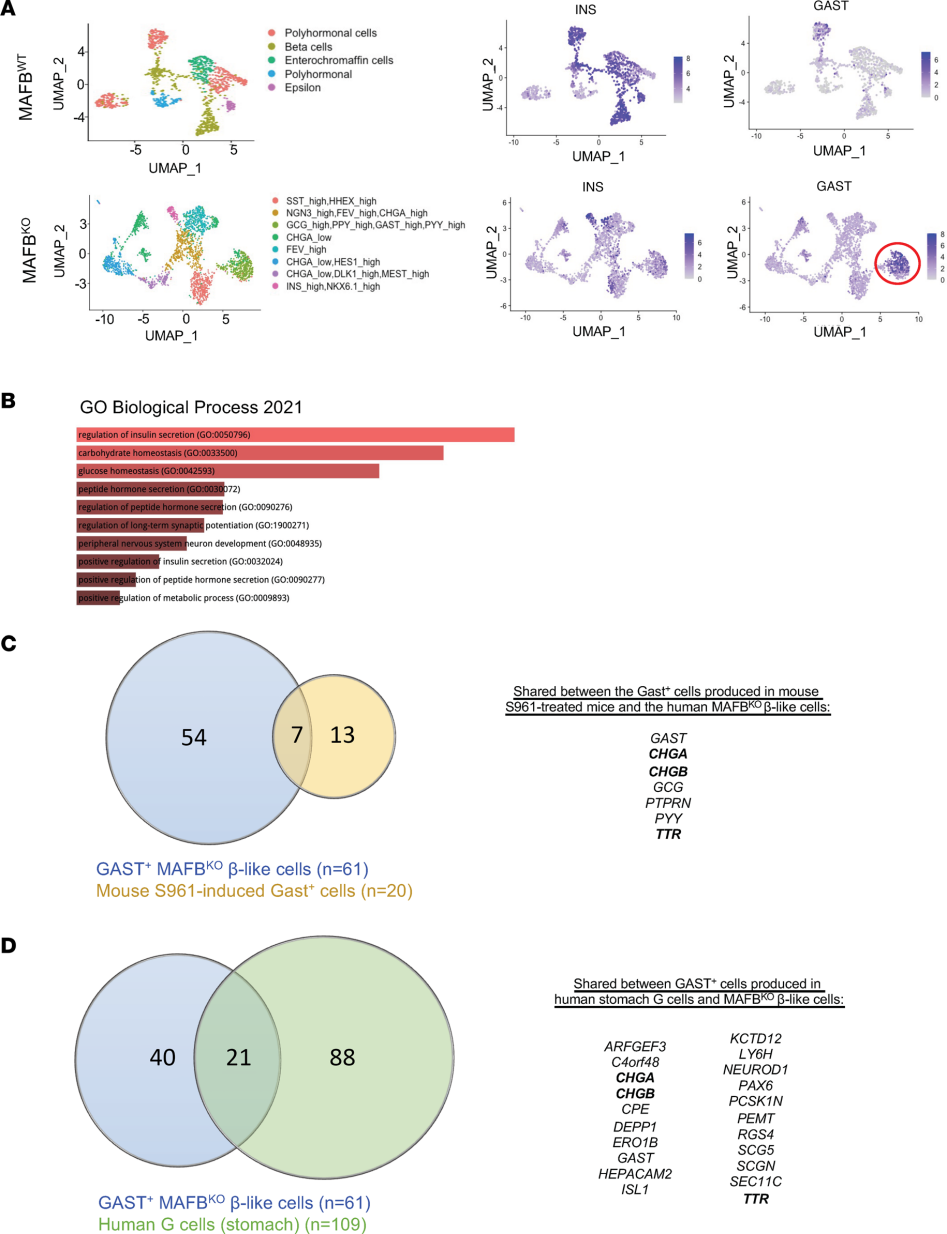
Unique and common gene signatures are produced in the Gast^+^ cells of mouse S961-treated β cells, hESC-derived MAFB^KO^ β-like cells, and bona fide stomach G cells. (**A**) UMAP of the single-cell RNA-Seq data from hESC-derived MAFB^WT^ and MAFB^KO^ cells from Russell et al. (8). Left panel: Cluster annotation (as determined by markers outlined in Supplemental Tables 7 and 8) identifies cell fates derived from this differentiation protocol. Right panel: Enrichment of GAST^+^ cells (encircled) and compromised INS^+^ cell numbers in the MAFB^KO^ cell population relative to MAFB^WT^ cells. (**B**) Top 10 most influenced GO biological processes based on the 61 upregulated genes in GAST^+^ MAFB^KO^ cells compared with GAST^–^ MAFB^KO^ cells. (**C** and **D**) Venn diagrams of DEGs comparing single-cell data from GAST^+^ cells from hESC-derived MAFB^KO^ β-like cells and Gast^+^ β cells of mouse S961-treated islets (**C**) as well as human stomach G cells (**D**). Gene products common to both conditions are listed on the right. Bolded gene products indicate those common to all 3 data sets.

By single-cell RNA-Seq analysis, 61 genes were found to be enriched in the GAST^+^ population of MAFB^KO^ β-like cells derived from hESC cells (Figure 4A and Supplemental Table 2). By gene ontology (GO) pathway analysis, many of these genes were linked to insulin (or hormone) secretion and glucose homeostasis (Figure 4B). Roughly 33% of the mouse S961-induced Gast^+^ cell signature overlapped with this human gene signature (7 of 20 genes; Figure 4C), while 34% of this human (21 of 61 genes) data set overlapped with genes enriched in bona fide GAST^+^ human stomach G cells (i.e., 109 genes; Figure 4D) (32). However, only 3 of the 61 genes were shared among these 3 populations (i.e., ~5%; CHGA, CHGB, and TTR; Figure 4, C and D). Consequently, there are both common and unique gene signatures found upon comparing the Gast^+^ cells generated pathologically from mouse β cells with human MAFB^KO^ GAST^+^ cells or normal human stomach G cells.

**Figure 5:**
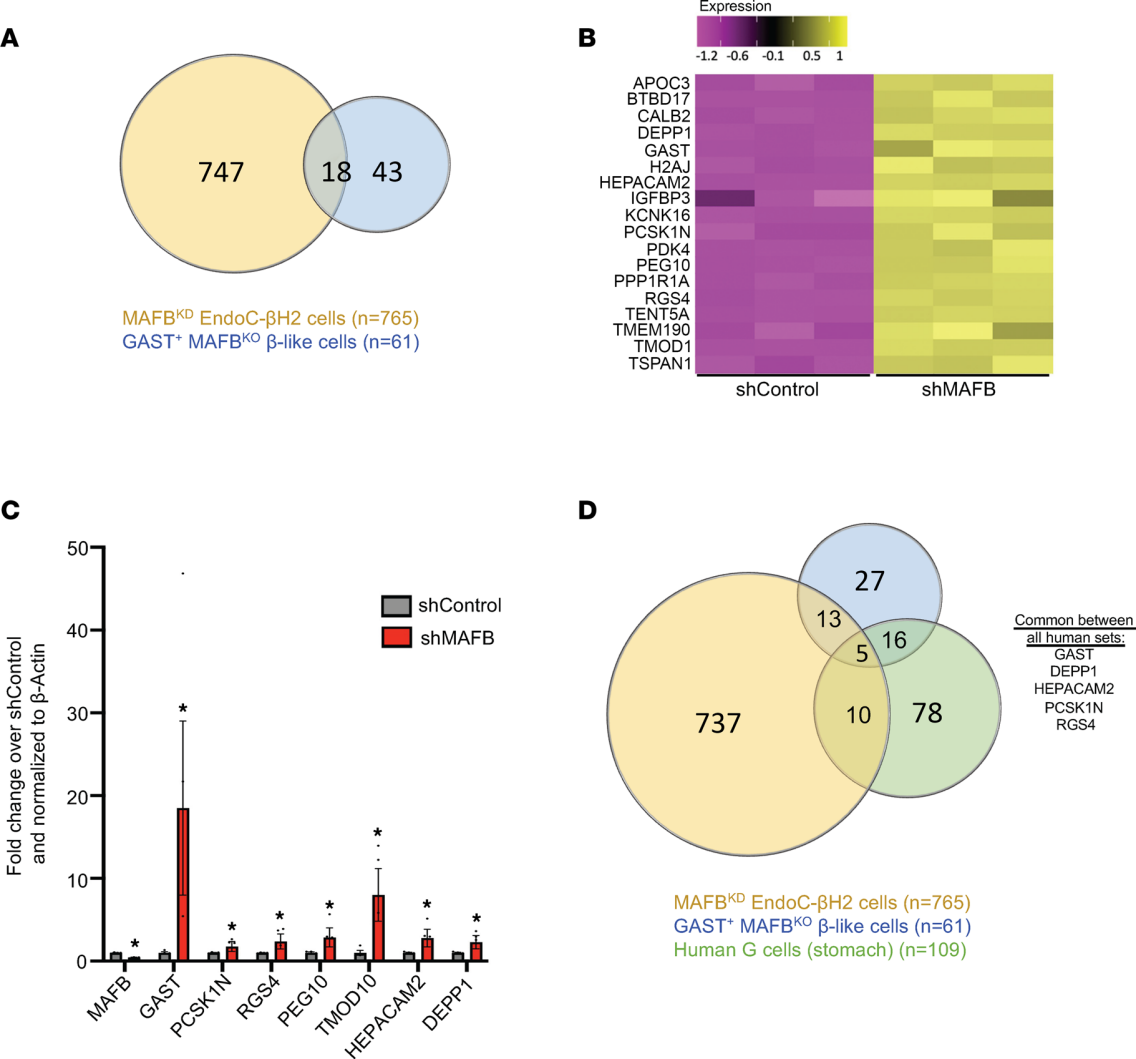
GAST^+^ MAFB^KO^ cell genes upregulated in MAFB^KD^ EndoC-βH2 cells. (**A**) Venn diagram of DEGs showing that 18 upregulated genes are shared between MAFB^KD^ EndoC-βH2 cells and the GAST^+^ MAFB^KO^ hESC–derived β-like cells. (**B**) Heatmap of these 18 upregulated shared genes in shMAFB- and shControl-treated EndoC-βH2 cells. *n* = 3 replicates/group. (**C**) qPCR analysis of a subset of the shared genes in shMAFB- and shControl-treated EndoC-βH2 cells. β-Actin served as the internal control and expression normalized to levels of shControl. Data are shown as mean ± SEM. *P < 0.05 by Student’s *t* test. *n* = 2 replicates/experiment, and experiments were repeated 3 times. (**D**) Venn diagram of DEGs of GAST^+^ MAFB^KO^ β-like cells, MAFB^KD^ EndoC-βH2 cells, and human stomach G cells. The 5 genes common to all 3 data sets are listed on the right.

RNA-Seq of MAFB^KD^ EndoC-βH2 cells was performed next for comparison with the single-cell data sets from hESC-derived MAFB^KO^ GAST^+^ β-like cells, mouse S961-induced islet Gast^+^ cells, and human stomach G cells. Approximately 30% (18 of 61) of the enriched genes in MAFB^KO^ GAST^+^ β-like cells were also found within the broader MAFB^KD^ EndoC-βH2 cell upregulated gene set, several genes of which were validated by quantitative PCR (qPCR) (Figure 5, A–C). However, just 14% (15 of 109) of MAFB^KD^ EndoC-βH2 enriched genes overlapped with those found in human G cells (Figure 5D), with simply *DEPP1*, *HEPACAM2,* *PCSK1N*, and *RGS4* present in all 3 human GAST^+^ cell contexts (Figure 5D). Interestingly, nonhormone genes normally expressed in other human islet endocrine cell types, such as TMOD1 (γ and PP cells), PEG10 (α, γ, and PP cells), PCSK1N (α and PP cells), and HEPACAM2 (δ, γ, PP cells) (33), were produced in both human GAST^+^ MAFB^KO^ β-like cells and MAFB^KD^ EndoC-βH2 cells. In fact, *PCSK1N*, *HEPACAM2*, and *RGS4* are also expressed in human stomach G cells (Figure 5D). Remarkably, *GAST* was the only common signature gene between the data sets from mouse S961-derived Gast^+^ cells, MAFB^KD^ EndoC-βH2 cells, hESC GAST^+^MAFB^KO^ β-like cells, and human G cells (Supplemental Figure 6). Together, our data not only demonstrate significant molecular diversity between normal GAST^+^ G cells and this hormone-producing cell population generated under pathophysiological conditions in the islet between species, but also highlight the importance of mouse MafA and human MAFB in regulating such genes within the broader islet β cell population.

### GAST^+^ cells are present in human islets deficient in MAFB.

**Figure 6:**
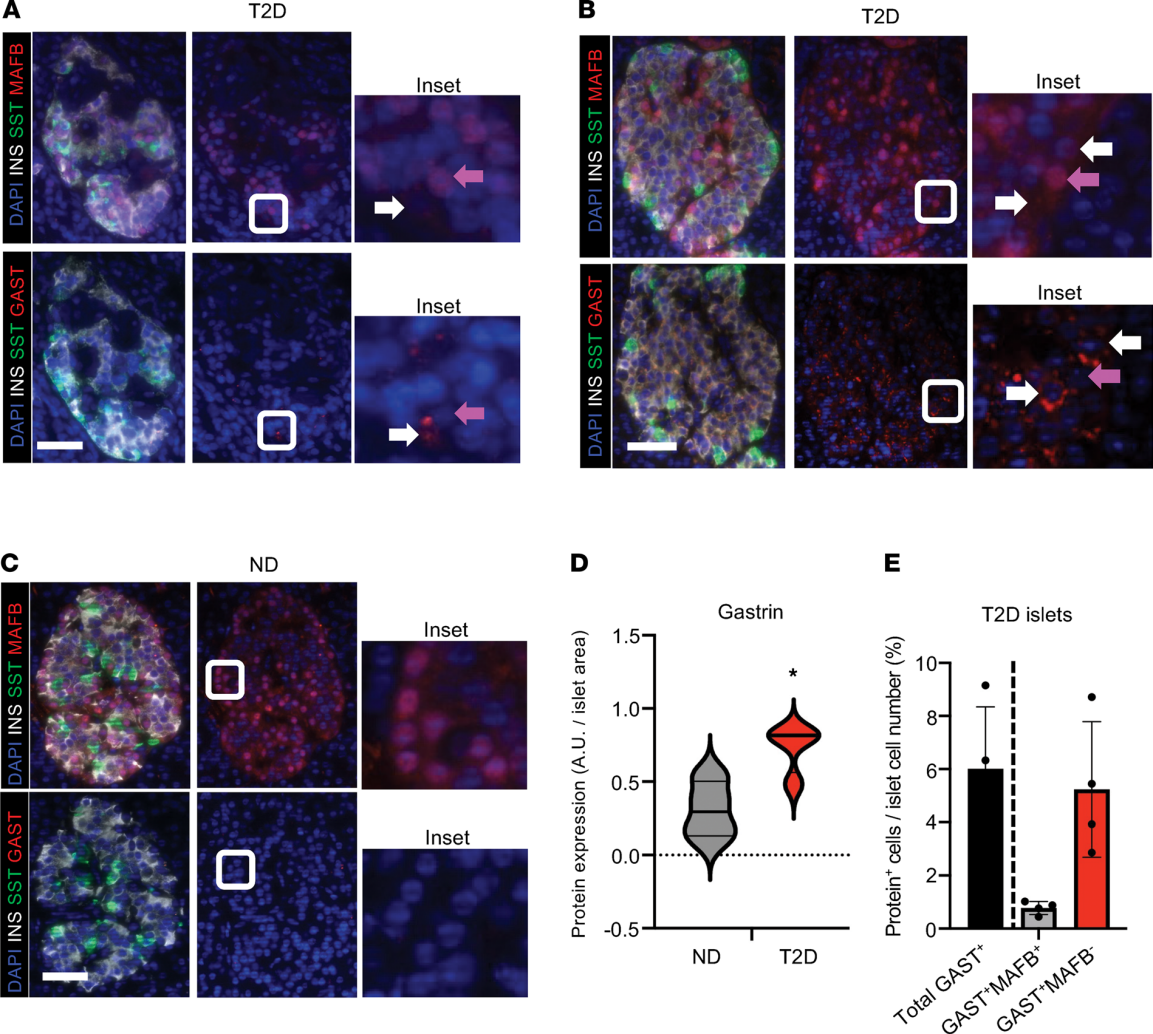
GAST is produced in MAFB-deficient T2D islet cells. (**A**–**C**) Representative images of immunostaining performed on serial sections from male age-matched diabetic (**A** and **B**) and nondiabetic (ND) (**C**) pancreata. MAFB, red in top panels; GAST, red in bottom panels; SST, green; INS, white; and nuclei, blue. GAST^+^ cells were not detected in healthy donor islets by immunofluorescence analysis (**C**). However, GAST production showed interislet variability and was associated with lower MAFB levels in T2D islet cells (**A** and **B**). Insets show magnified view of representative GAST^–^MAFB^+^ (purple arrows) and rare GAST^+^MAFB^–^ (white arrows) cells. *n* = 4 age- and sex-matched donors/condition. Scale bar, 100 μm. (**D**) Quantification of GAST signal (A.U.) per islet between ND and T2D male donors. Data are shown as mean ± SEM. *P < 0.05 by Student’s *t* test. *n* = 4 age- and sex-matched donors/condition, 4–6 islets per donor. (**E**) Quantification of GAST and MAFB colocalization in T2D male donor islets (% islet cells). Data are shown as mean ± SEM. *n* = 4 age- and sex-matched donors/condition, 4–6 islets per donor. Donor information in Supplemental Table 3.

Both MAFA and MAFB are highly sensitive to glucotoxicity and oxidative stress, with protein levels reduced in T2D islets (34, 35). To determine if MAFB levels were low in the GAST^+^ cells produced in T2D islets (31), serial sections from age- and sex-matched normal and T2D donors were immunostained for both proteins (Supplemental Table 3). Indeed, enrichment of GAST^+^ cells were found in MAFB-deficient T2D islets (Figure 6, A–E). GAST^+^ cells appeared to also be produced in female T2D samples (Supplemental Figure 7).

To further analyze the relationship between human *GAST* gene expression and MAFB, *GAST* mRNA levels were measured in human islets following either a nonselective U6- or rat insulin II enhancer/promoter-driven lentiviral-mediated *MAFB* knockdown (pseudoislets). *GAST* was elevated after a roughly 50% *MAFB* knockdown in these contexts in 2 ND donors (Supplemental Figure 8 and Supplemental Table 3). These results further support a role for MAFB in repressing *GAST* expression in adult human islet β cells.

### MAFB directly regulates human GAST transcription.

**Figure 7:**
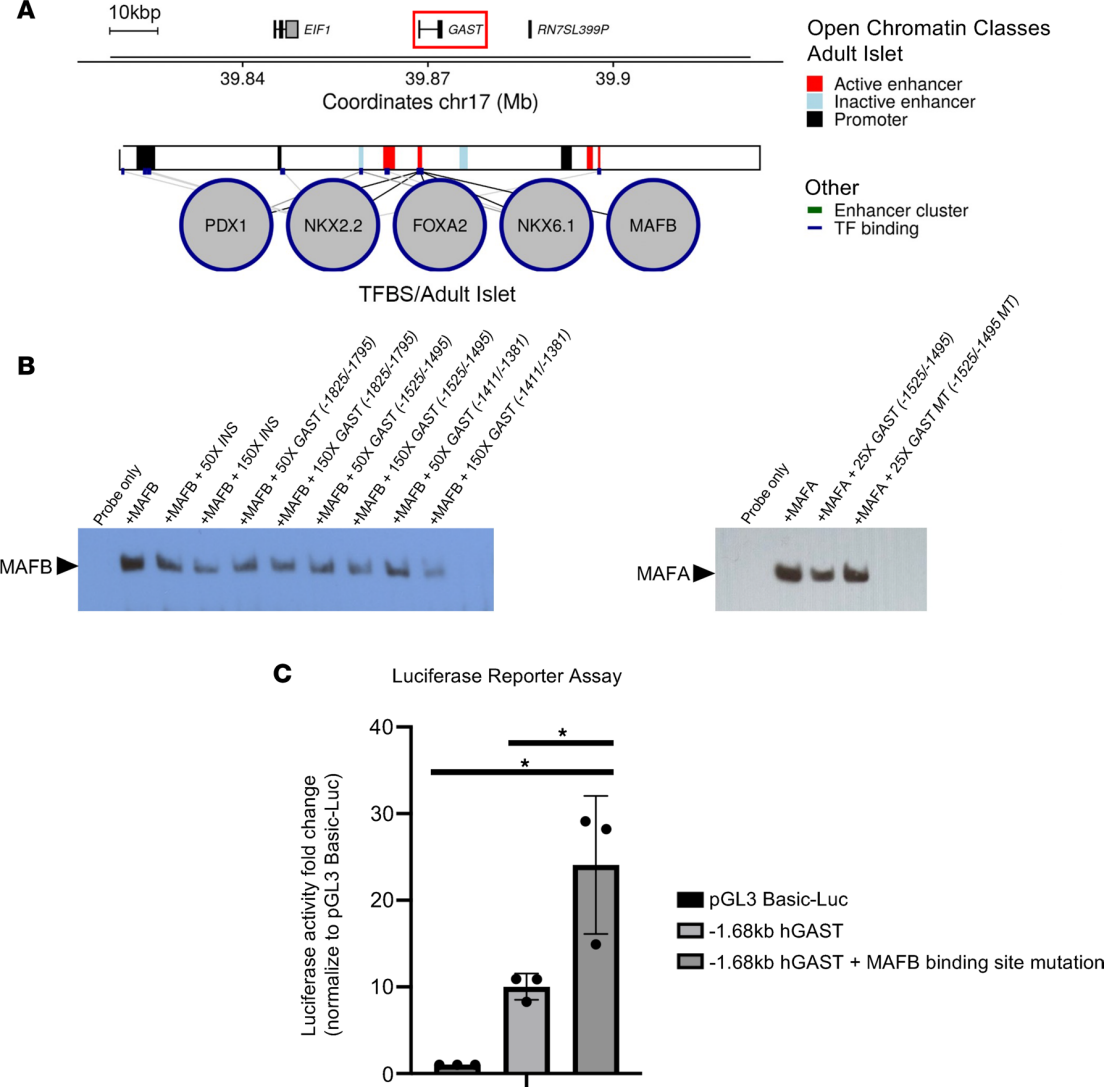
MAFB binds within the human *GAST* 5′-flanking region and suppresses gene expression in β cells. (**A**) ChIP-Seq data derived from whole islets by Pasquali et al. (36) illustrating islet-enriched PDX1, NKX2.2, FOXA2, NKX6.1, and MAFB TF binding sites (TFBS) within approximately 1.5 kbp of the human islet *GAST* gene transcription start site. (**B**) Gel shift analysis of MAFB (left) or MAFA (right) protein binding to a biotin-labeled human *INSULIN* (*INS*) probe to a MAFA/B binding site starting at position –135 in the presence of human *INS* and human *GAST* –1,825/–1,795, –1,525/–1,495, or –1,411/–1,381 bp unlabeled competitors. While all 3 *GAST* sites competed as effectively as the *INS* element, only the *GAST* –1,525/–1,495 element is conserved in mouse and also binds MAFA (right panel). *n* = 3 experimental replicates. (**C**) Representative MAFA/B binding site mutation in the –1,525/–1,495 element stimulated –1.68 kbp *GAST*-driven luciferase reporter activity in human EndoC-βH1 cells. pGL3 Basic-LUC represents the control vector without insert. Data are shown as mean ± SEM. *P < 0.05 by 2-way ANOVA. *n* = 3 experimental replicates, and experiments were repeated 3 times.

Because human islet MAFB binding sites were detected approximately 1.5 kb upstream of the *GAST* gene transcriptional start site by ChIP-Seq (36, 37), we asked whether this TF could directly bind and control β cell gene transcription (Figure 7A). Notably, the presence of other islet-enriched TF binding sites (i.e., those of PDX1, NKX2.2, FOXA2, and NKX6.1) in this region indicated that these may also act in concert with MAFB to negatively or positively affect expression (36).

The ability of MAFB to bind to 5′-flanking sequences in the human *GAST* gene was analyzed in gel mobility shift assays using CMV-driven MAFB plasmids transfected HeLa nuclear extracts. This region of *GAST* appears to contain 3 MAFB binding sites (i.e., –1,825/–1,795, –1,525/–1,495, and –1,411/–1,381), as indicated by their ability to compete effectively in relation to a well-characterized MAFA/MAFB binding site in the human *INS* gene (Figure 7B, left). Only one of these human MAFB binding sequences is conserved in mice (i.e., –1,525/–1,495), and it also effectively bound to MAFA (Figure 7B, right). As predicted for a repressor binding site, mutation of this conserved element prevented the inhibitory effect of MAFB and led to elevated expression of the –1.68 kb–driven *GAST* reporter in transfected EndoC-βH1 cells (Figure 7C). Moreover, expressing MAFB in MAFB^KD^ EndoC-βH2 cells attenuated *GAST* induction to near-baseline levels, further supporting this TF’s role in suppressing *GAST* expression (Supplemental Figure 9).

## Discussion

MAFB was not only shown to be essential for insulin production during the formation of hESC-derived β-like cells but also in preventing non–β cell hormone expression (8). Here, we analyzed if similar regulatory properties were found for MafA during the formation of mouse islet β cells, and MAFA and/or MAFB in adult human β cells. To this end, we compared the gene regulatory properties of these TFs across multiple conditions — including direct genetic manipulation of MafA in mouse islet β cells, MAFA^KD^ and MAFB^KD^ in human EndoC-βH1/2 β cells, MAFB^KO^ in hESC-derived β-like cells, and MAFB^KD^ in human pseudoislets as well as in pathophysiologic conditions — in mouse models and human T2D islets. Our experimental results revealed that: (a) the genes activated by insulin resistance in Gast^+^ β cells are also expressed in the mouse *MafA*^∆β^ and *ob/ob* islet β cell population and not in normal islet α, δ, or PP cells; (b) MAFB^KD^, but not MAFA^KD^, induces GAST^+^ cell production in EndoC-βH1/2 cells, demonstrating species-specific regulation by these TFs; (c) the GAST^+^ cells produced in MAFB^KO^ hESC–derived β-like cells or pathologically in mouse models are distinct from physiologic human stomach G cells; (d) readily detectible Gast protein production is restricted to a small subpopulation of MafA/MAFB-deficient β cells even though the genes enriched in these cells are present in the broader TF-depleted β cell population; and (e) MafA and MAFB appear to directly repress *GAST* transcription in mouse and human β cells, respectively. Since the levels of MAFA and MAFB are more sensitive to metabolic stressors in relation of other islet-enriched TFs (34), we propose that compromised MAF expression upon exposure to obesity- and insulin resistance–induced effectors leads to β cell dysfunction and reduced cell identity due to induction of GAST^+^ cell–associated gene products. These events may precede β cell dedifferentiation and T2D development.

Of the many peptide hormones expressed by the mouse pancreatic islet, Gast is uniquely restricted to the fetal pancreas. Pancreatic Gast^+^ cells are derived from Ngn3 TF^+^ endocrine progenitors, which are abundant during development but wane at birth, and are undetectable in adult pancreata (31). Deletion of *Gast* from the mouse pancreas does not affect islet cell composition or the islet proliferative response to injury (38). However, Gast has been proposed to represent an endocrine cell reprogramming marker, with production representing a potentially reversible cell state (21). Consequently, GAST^+^ cells may represent a pool of “reprogrammable” cells of loosened identity that can be coaxed back to β cells or produce new β cells from non–β cell sources in an autocrine and/or paracrine manner. Several groups have attempted to harness its properties to expand the β cell compartment (39–44); thus, it is prudent to understand how similar GAST^+^ cells produced either from human MAFB^KO^ hESC β-like cells or pathophysiologically in the setting of MAFB deficiency are to bona fide, physiologic human GAST–expressing cells. While the molecular composition of embryonic GAST^+^ cells of the pancreas is unknown, there was ~33% overlap of the enriched gene signature between the GAST^+^ cells generated from human MAFB^KO^ hESC β-like cells to human stomach G cells, and to mouse S961-treated β cells (Figure 4, C and D). However, very few of these genes are shared between the mouse and human GAST^+^ cell populations (i.e., 5%; Figure 4, C and D). Hence, even though the GAST^+^ cells produced by insulin resistance in mice or the MAFB^KO^ GAST^+^ β-like cells have some molecular similarities to stomach G cells, these conditions alone are insufficient to completely reprogram the β cell toward a de facto G cell. Nonetheless, the loosened β cell identify caused by expression of genes shared among GAST^+^ cells induced by stress, insulin resistance, and MafA and/or MAFB deficiency may represent markers of a reversible cell state.

The heterogeneity among Gast^+^/GAST^+^ cells produced in mouse *MafA*^∆β^ islets, human MAFB^KD^ EndoC-βH1/2 cells, and human G cells could reflect a variety of factors. For example, baseline β cell heterogeneity in MafA/MAFB and calcium/calcineurin signaling may contribute, as deficiency in both are involved in GAST^+^ β cell production (31). In addition, this heterogeneity could entail experimental context (i.e., animal versus cell line) and/or differences in temporal induction of Gast^+^ cell–associated genes in relation to loss to TF expression.

Strikingly, Gast protein was only detected in a small number of cells in *MafA*^∆β^ mouse islets (Figure 1C), S961-treated mouse islets, MAFB^KD^ EndoC-βH2 cells (Figure 3C), and T2D islets (Figure 6) in single-cell analyses. Consequently, MAFB alone is not sufficient to induce *GAST* expression within the general β cell population, and stochastic/redundant regulation exists to maintain cell fidelity. This work reveals questions about the regulatory events leading to β cell dysfunction and loss of identity upon exposure to the prediabetic stress conditions associated with insulin resistance and obesity. The involvement of MafA and MAFB is supported by the loss of MafA in obese mouse models (45–47), high sensitivity of MAFA and MAFB to various islet cell stressors (e.g., high glucose and cytokines; refs. 48, 49), their reduction in T2D islets (34, 50), and compromised GSIS with MAFB depletion in human EndoC-βH1 cells (6) and islets (35). It is also noteworthy that detrimental effects on adult β cell activity caused by MafA or MAFB reduction are much subtler than found for other islet-enriched TFs, many of which are associated with maturity-onset diabetes of the young (51, 52). Accordingly, our data indicate that prediabetic β cell dysfunction is (at least in part) due to loss of mouse MafA and human MAFB as well as induction of a Gast^+^/GAST^+^ cell–like molecular phenotype.

Importantly, it is possible that continual repression of non–β cell transcriptional programs is another less-appreciated function of islet-enriched TFs that are presently associated with gene activation. For example, deficiency in other key islet TFs permit misexpression of non–β cell hormones, such as Gcg, Sst, Ppy, Pyy, and Ghrelin. These results reveal the significance of such TFs in controlling intraislet cell plasticity (reviewed in ref. 12). However, Gast^+^ cells were not produced upon downregulation of Pdx1 (53), Nkx6.1 (54), Nkx2.2 (55), or Pax6 (25), indicating distinct regulation of cell identity by such TFs (12). It is noteworthy that MafA mutant mice have a subtler islet phenotype than these other islet-enriched TF mutants (56), correlating with earlier inducers of β cell dysfunction such as obesity. Moreover, factors mediating human islet gene dysregulation are not well established. Here, we identify a direct regulation by MAFB (and not MAFA) of *GAST* misexpression seen in T2D.

More robust *Gast* and *Cck* expression was identified in male than female mouse islets in hyperglycemic mice and *MafA*^∆β^ mice, suggesting a greater vulnerability of male β cells to metabolic stressors and alternative cell states. This is supported by the higher rates of overt diabetes in males than females in mouse models of diabetes and multiple forms of human diabetes in specific populations (reviewed in ref. 57). In addition, transplantation of hESC-derived β-like cells into female recipient mice often yield faster in vivo maturation and insulin secretion properties than male recipient mice (58). Interactions between the MafA, MAFB, and nuclear sex hormone receptors (i.e., estrogen receptor β and androgen receptor) warrant further study, especially given the results described here and the sex-dependent differences in β cell gene expression found in normoglycemic mice (59) and by production of the S64F MAFA variant (60).

Although the presence of GAST^+^ cells in human T2D islets was recognized earlier (31), the relevance to diabetes pathogenesis and mechanism was unclear. Our results strongly suggest that GAST^+^ gene signatures are enriched in metabolically stressed, but prediabetic, islet β cells. Moreover, the enrichment of these signature genes throughout an islet population with a limited number of overt GAST^+^ cells further implicate an increased sensitivity to MafA or MAFB protein levels. As a result, we predict that these associated gene signatures will be induced even earlier than GAST itself upon pathologically induced loss of MafA or MAFB, which could be tested temporally in future studies in models of insulin resistance (i.e., following S961 or high-fat diet treatment). It is also unclear how these differentially expressed genes influence islet β cell dysfunction or identity under these conditions. Additionally, we recognize that several of the islet GAST^+^ cell enriched signature genes are normally expressed in other islet endocrine cells; thus, determining if differences in their regulation occur will require detailed investigation across normal, prediabetic, and diabetic settings using β cell–enriched experimental methods. In fact, GAST^+^ δ cells have been detected in human T2D, although MAFB is not typically expressed in this cell type. Here, we find that SST is induced in a MAFB-deficient, pure β cell line (Figure 3C); however, we cannot exclude the possibility of δ cell reprogramming in an in vivo setting. Along this line, MAFB is robustly expressed in islet α cells, which raises the question how the compromised levels of this TF in pathogenic settings (e.g., T2D; ref. 34) affects these cells**.**

In summary, our results highlight alternative β cell states imposed by metabolic stressors. This work puts forth a potentially novel regulatory role of islet MafA and MAFB in maintaining monohormonal β cell identity by actively repressing non–β Gast^+^/GAST^+^ cell–associated transcriptional programs. Loss of MAFB in early T2D may promote β cell reprogramming; thus, understanding the stepwise transitions toward dysfunction, which are potentially reversible, may facilitate the production of therapies in which early β cell dysfunction can be targeted to slow the progression to overt diabetes. Future studies to understand the natural history and production of GAST^+^ and other non–β cell hormone^+^ cells (e.g., CCK), under physiologic and pathophysiologic conditions, and identifying strategies to target these cells will be critical in our understanding of alternative β cell fates and potentially coaxing their return to normal functional β cells.

## Methods

### MafA^Δβ^ and ob/ob mice generation and S961 treatment in mice.

*MafA*^Δβ^ mice were generated by crossing *MafA^fl/fl^* (3) with *Ins1*^Cre^ (*Ins1*^tm1^ ^Cre^) knockin mice (16). *MafA^fl/fl^* *Ins1*^Cre^ mice were then crossed with *Ins2^Apple^* mice (61). For isolation of β cells, islets from 3-month-old Apple^+^ male mice were sorted by FACS on the red spectra to an average purity of 85%–95%. *Gast*^WT^ and *Gast*^KO^ mice were originally generated by targeted disruption of the *Gast* gene (62, 63), and stomach tissues collected at 8 weeks of age were provided as a gift from Eunyoung Choi and James Goldenring (Vanderbilt University Medical Center, Nashville, Tennessee, USA). *Ob/ob* mice (The Jackson Laboratory) were crossed with *mIns1-H2b-mCherry* reporter expressing mice (64), and the β cells from 35- to 38-week-old male and female lean and *ob/ob* mouse islets were sorted by FACS based upon their mCherry production. The S961-treated insulin-resistant mouse model (31) was generated by either vehicle (PBS, Corning) or 12 nmol S961 loading into an ALZET osmotic pump and implanted s.c. on the back of 6-week-old male ICR (CD-1) mice for 4 days. The S961 insulin receptor antagonist was a gift from Novo Nordisk to the Yuval Dor Lab (The Hebrew University-Hadassah Medical School, Jerusalem, Israel).

### Single-cell RNA-Seq and analysis of S961-treated mouse islets.

Control and S961-treated diabetic mouse islets were isolated, dissociated, and stained with propidium iodide (PI, Thermo Fisher Scientific). Live cells (PI^–^) were sorted using a FACSAria III (BD Biosciences). Libraries were made using Chromium Single Cell 3′ Reagent Kits V3 (10X Genomics) following the manufacturer instructions and sequenced on an Illumina HiSeq 2500. Raw reads of each sample were processed using the count command of the Cell Ranger software (v2.0.2), aligning the reads to the mouse mm10 (GRCm38) genome version. The generated report was used to assess the quality of the samples in terms of cell numbers (3,072 [PBS], 2,443 [S961]), average reads per cell (104,108 [PBS], 149,852 [S961]), fraction of reads in cells (92.3% [PBS], 93.6% [S961]), alignment rate, and saturation (57.9% [PBS], 61.3% [S961]). The PBS- and S961-treated groups were further filtered to remove doublets and leave only high-quality cells as previously described (65, 66). Data sets were normalized using LogNormalize, a global-scaling normalization method. Cluster annotation was performed as previously described (66). The Seurat FindMarkers function using the nonparametric Wilcoxon rank-sum test was used for the identification of differentially expressed genes between clusters within and between the experimental groups, with log_2_ fold change threshold and the minimum fraction of gene detection set to 0. Figures to visualize the clusters and the marker expression in the low-dimensional space (uniform manifold approximation and projection [UMAP]) were generated by Seurat functions. Expression of genes enriched in Gast^+^ cells are listed in Supplemental Table 1. Sequencing data were deposited in the National Center for Biotechnology Information (NCBI) Gene Expression Omnibus (GEO) database (accession no. GSE234770).

### Bulk RNA-Seq and analysis of MafA^∆β^ mouse β cells, ob/ob mouse islet β cells, and human EndoC-βH2 cells.

The RNeasy Micro Plus Kit (QIAGEN) was used to isolate total RNA from EndoC-βH2 cells, FACS-purified *MafA*^∆β^ mouse islet β cells, and lean and *ob/ob* male and female β cells sorted by FACS (*n* ≥ 3 independent replicates). Isolated RNA quality was analyzed on an Agilent 2100 Bioanalyzer. The cDNA libraries were constructed, and paired-end sequencing was performed on an Illumina NovaSeq6000 (150-nucleotide reads). The generated FASTQ files were processed and interpreted using the Genialis visual informatics platform (https://www.genialis.com) as described previously (60). DESeq was used for differential gene expression analyses and statistical comparison, as previously described (64). Poorly expressed genes, which have expression count summed over all samples below 10, were filtered out from the differential expression analysis input matrix. Sequencing data were deposited in the NCBI GEO database (GSE230728 for *MafA*^∆β^ mouse β cells; GSE224797 for *ob/ob* mouse β cells; and GSE228992 for EndoC-βH2 cells).

### In silico single-cell RNA-Seq analysis of mouse islet cell types, MAFB^KO^ hESC β-like cells, and human Gast^+^ G cells.

For mouse islet cell types, raw gene count matrices were extracted from existing mouse single-cell RNA-Seq data sets (67–69). The sequencing data used can be found in the NCBI GEO database (accession nos. GSE84133, GSE77980, GSE109774). Quality control measures were taken as described (70), and source code is provided to merge data sets and distinguish islet cell types. Seurat analyses were used to visualize the results. The sequencing data used for hESC β-like cells can be found in the NCBI GEO database GSE145347 (8). Cell clustering analysis in MAFB^WT^ and MAFB^KO^ hESC β-like cells was performed using markers listed in Supplemental Tables 7 and 8, respectively. Sequencing data for human G cells is available in the NCBI GEO database GSE134520. The marker genes enriched in primary human Gast^+^ G cells were identified using the FindAllMarkers function with settings on genes with at least 2-fold higher compared with remaining cells and provided within the original report (32). The gene marker list generated for each of these data sets (mouse islet cell types, hESC β-like cells, and human G cells) were overlaid with the mouse Gast^+^ and human GAST^+^ islet signatures (Supplemental Tables 1 and 2) for comparison.

### Islet isolation, lentivirus-mediated shRNA-based knockdown and overexpression, RNA isolation, cDNA synthesis, and real-time PCR.

Mouse islets were isolated using collagenase P (Roche) injected into the pancreatic duct, followed by a Histopaque (1077; Sigma-Aldrich) gradient. Islets were incubated overnight in standard RPMI-1640 medium (Thermo Fisher Scientific) supplemented with 10% FBS, L-glutamine, and penicillin-streptomycin in a 5% CO_2_ incubator at 37°C. Hand-picked islets were subject to RNA isolation and qPCR analysis. Knockdown experiments were performed in EndoC-βH1/2 cells (29, 30). The rat insulin II enhancer–driven shMAFB (i.e., shMAFB-RIP) construct was generated by subcloning the MAFB targeting sequence into a CMV-RIP vector (71), a gift from David Jacobson (Vanderbilt University, Nashville, Tennessee, USA). Plasmids from scrambled shRNA (targeting sequence: CCTAAGGTTAAGTCGCCCTCG; VectorBuilder, VB190410-1184rda), MAFA shRNA (targeting sequences: 5′-TAATAATTATAAATACTTCGAA-3′ and 5′-TAAAACAAGATGTATTTCCCCA-3′; GeneCopoeia), MAFB shRNA (targeting sequence: 5′-GCCCAGTCTTGCAGGTATAAA-3′; VectorBuilder, VB180215-1122jqv), and MAFB overexpression (VectorBuilder, VB220317-1298cwn) were packaged into lentiviruses employing human embryonic kidney 293T cells and concentrated using the PEG-it Virus Precipitation Solution (System Biosciences), as previously described (8). Cells were infected for 36 hours, followed by 1:5,000 puromycin selection for 36–48 hours, with the selection time point dictated by the GFP signal from the infecting virus. Mouse islet RNA was then isolated using the RNeasy Micro Kit (QIAGEN), while human cell RNA was collected using the TRIzol reagent (Invitrogen) and a DNA-free RNA kit (Zymo Research). cDNA was generated using Superscript III reverse transcriptase (Invitrogen) by the oligo(dT) priming method. Real-time PCR assays were performed using the LightCycler FastStart DNA Master PLUS SYBR Green kit (Roche) and a LightCycler PCR instrument (Roche). The real-time PCR primers are listed in Supplemental Table 4. *Gapdh* or *β-Actin* was used to normalize the data. Real-time PCR results were analyzed using the ΔΔCt method.

### Human pancreatic specimens.

Pancreata from nondiabetic (ND) and T2D donors were obtained by the Powers-Brissova laboratory through partnerships with the International Institute for Advancement of Medicine (IIAM), National Disease Research Interchange (NDRI), local organ procurement organizations (OPO), Network for Pancreatic Organ Donors with Diabetes (nPOD), and Imagine Pharma Islet Center. Donor information is detailed in Supplemental Table 3. Deidentified medical histories describe disease status as well as clinical characteristics.

### Human pseudoislet generation.

Primary human islets were acquired from the Integrated Islet Distribution Program (IIDP) and Human Pancreas Analysis Program (HPAP) and cultured in CMRL 1066 (MediaTech, 15-110-CV media [5.5 mM glucose], 10% FBS [MilliporeSigma, 12306C], 1% penicillin/streptomycin [Thermo Fisher Scientific, 15140-122], 2 mM L-glutamine [Thermo Fisher Scientific, 25030-081]) in 5% CO_2_ at 37°C for approximately 24 hours before beginning the studies. Briefly, human islets were handpicked to > 95% purity and then dispersed with 0.025% HyClone trypsin (Thermo Fisher Scientific). Islet cells were counted and infected with the shRNA lentivirus at an MOI of 500. Infected cells were seeded at 2,000 cells per well in Cell-Carrier Spheroid Ultra-low attachment microplates (PerkinElmer) in enriched Vanderbilt pseudoislet media (72). Cells were allowed to reaggregate for 6 days before being harvested for RNA isolation and qPCR analysis. Islet donor information is listed in Supplemental Table 3.

### EndoC-βH1 and EndoC-βH2 cell maintenance and analysis.

The human cell lines were maintained under conditions described previously (29, 30). The growth medium contains DMEM (Thermo Fisher Scientific), 5.6 mmol/L glucose (MilliporeSigma), 2% BSA Fraction V (Roche), 100 U/mL penicillin (Gibco), 100 μg/mL streptomycin (Gibco), 50 mmol/L 2-mercaptoethanol (MilliporeSigma), 10 mmol/L nicotinamide (Thermo Fisher Scientific), 5 mg/mL transferrin (MilliporeSigma), and 6.7 ng/mL sodium selenite (MilliporeSigma). EndoC-βH1/2 cells were infected for 1–2 weeks with 150 ng shControl, shMAFA, or shMAFB lentiviral particles per million cells before harvesting for immunoblotting and qPCR analysis. Knockdown of human NCOA6 was performed by siRNA transfection (6) (Dharmacon) for 48 hours before harvesting for qPCR analysis. MAFA and MAFB protein levels were normalized to endogenous β-actin by immunoblotting with anti-MAFA (Cell Signaling Technology, 79737), anti-MAFB (MilliporeSigma, HPA00563), and anti–β-actin (MilliporeSigma, MAB1501) antibodies. Horseradish peroxidase–conjugated anti-rabbit (31460, Thermo Fisher Scientific) or anti-goat (31402, Thermo Fisher Scientific) secondary antibodies were used at 1:5,000. Immunoblots were quantitated with ImageJ software (NIH). Antibody information can be found in Supplemental Table 5. Human GAST protein from cell lysate was measured by high-sensitivity ELISA per manufacturer recommendations (LSBio, LS-F4489). The *GAST-*driven firefly luciferase constructs were transfected in EndoC-βH1 cells along with phRL-TK internal control (Promega) using the Lipofectamine protocol (Invitrogen). *GAST-*driven firefly luciferase spans sequences from –1,680 bp to +8 bp, and the –1,680 bp to +8 bp and –1,525/–1,495 element mutants were constructed in the pGL3-Basic luciferase reporter vector (Promega) using standard molecular biology techniques. The MafA/B binding site mutant was constructed using the Quickchange II site directed mutagenesis kit (Agilent), and mutagenesis was confirmed by DNA sequencing. Oligonucleotides used are provided in Supplemental Table 6. Cellular extracts were collected 48 hours after transfection, and the Dual-Luciferase Reporter Assay (Promega) was performed according to the manufacturer’s directions. Firefly luciferase measurements were normalized to the Renilla phRL-TK internal control.

### Electrophoretic mobility shift assay.

HeLa cells (MilliporeSigma) were transfected with either a CMV-driven MAFA or MAFB expression plasmid using the Lipofectamine protocol (Thermo Fisher Scientific). Forty-eight hours later, nuclear extract and DNA binding reactions were performed. Briefly, 10 μg of nuclear extract and 200 fmol of the biotin-labeled double-stranded human *INS* MAFA/B binding site probe were mixed either alone or with unlabeled competitor DNAs in a 20 μL reaction system (LightShift Chemiluminescent EMSA Kit, Thermo Fisher Scientific) containing 1***×*** binding buffer, 2.5% glycerol, 5 mM MgCl, 50 ng/μL of poly(dI-dC) (Thermo Fisher Scientific), and 0.05% NP-40 (Sigma). MAFA and MAFB antibody super-shift reactions were performed. The WT and mutant MAFA/B binding site sequences are provided in Supplemental Table 6. The reactions were separated on a 6% precast DNA retardation gel in 0.5% Tris borate-EDTA buffer (TBE, Thermo Fisher Scientific) at 100 V for 1.5 hours.

### Immunofluorescence staining and quantification.

Human pancreata, mouse pancreata, and treated EndoC-βH1/2 cells were fixed in fixed in 4% paraformaldehyde (Electron Microscopy Services) in PBS on ice (6-hour fixation for pancreata, 15-minute fixation on slides for cells), and pancreata were embedded in either Tissue-Plus OCT (Thermo Fisher Scientific) or paraffin wax. The sections of human and rodent pancreas as well as fixed EndoC-βH1/2 cells were made permeable by 0.5% Triton treatment for 10 minutes. The paraffin sections were deparaffinized and rehydrated before citrate buffer–based antigen retrieval. Following blocking with 0.5% BSA in PBS for 120 minutes, the primary antibodies were applied overnight at 4°C. Species-matched antibodies conjugated with the Cy2, Cy3, or Cy5 fluorophores were used for secondary detection (1:1,000; Jackson ImmunoResearch). DAPI was used for nuclear staining (Southern Biotech). Immunofluorescence images were obtained using the Zeiss Axio Imager M2 widefield microscope with ApoTome. Quantification of protein signals and nuclear count were performed by ImageJ analysis. Primary antibodies are listed in Supplemental Table 5.

### Statistics.

Data are expressed as the mean ± SEM. Statistical analysis was performed using GraphPad Prism 9.5.0 (GraphPad Software Inc.). The differences between groups were analyzed by unpaired 2-tailed Student’s *t* test or 2-way ANOVA, as indicated. Differences were considered to be statistically significant at *P* < 0.05.

### Study approval.

Mice of both sexes were used in this study; details can be found in the results section and figure legends. All animal studies were reviewed and approved by the Vanderbilt University IACUC. Mice were housed and cared for according to the Vanderbilt Department of Animal Care and the IACUC of Animal Welfare Assurance Standards and Guidelines. The Vanderbilt University IRB declared that studies on deidentified human pancreatic specimens do not qualify as human subject research.

### Data availability.

All data supporting the findings of this study and its supplementary information files are available within the article and can be accessed from public repositories or in Supporting Data Values. Bulk and single-cell RNA-Seq data sets newly generated in this paper have been deposited in the GEO database under the accession nos. GSE234770 for S961-treated mouse β cells, GSE230728 for *MafA*^∆β^ mouse β cells, GSE224797 for *ob/ob* mouse β cells, and GSE228992 for EndoC-βH2 cells.

## Author contributions

JC, XT, EMW, and RS designed the study. JC, XT, EMW, TD, VAC, SA, RR, ABO, AMM, MG, JL, and ZAL performed experiments. JC, XT, EMW, TD, VAC, SA, RR, ABO, and AMM performed bioinformatic analysis. JC, XT, EMW, TD, VAC, SA, RR, ABO, AMM, MG, JL, ZAL, MOH, MAM, MH, YD, and RS analyzed data. JC, XT, and RS wrote the manuscript. JC and XT are co–first authors due to their contributions in most of the experiments described. JC was listed first for having a larger role in writing the manuscript.

## Supplementary Material

Supplemental data

Supplemental tables 1-7

Supporting data values

## Version 1 08/22/2023

Electronic publication

## References

[1] Hang Y, Stein R (2011). MafA and MafB activity in pancreatic β cells. Trends Endocrinol Metab.

[2] Pan FC, Wright C (2011). Pancreas organogenesis: from bud to plexus to gland. Dev Dyn.

[3] Artner I (2010). MafA and MafB regulate genes critical to beta-cells in a unique temporal manner. Diabetes.

[4] Cyphert HA (2019). Examining how the MAFB transcription factor affects islet β-cell function postnatally. Diabetes.

[5] Banerjee RR (2016). Gestational diabetes mellitus from inactivation of prolactin receptor and MafB in islet β-cells. Diabetes.

[6] Scoville DW (2015). MLL3 and MLL4 methyltransferases bind to the MAFA and MAFB transcription factors to regulate islet β-cell function. Diabetes.

[7] Hang Y (2014). The MafA transcription factor becomes essential to islet β-cells soon after birth. Diabetes.

[8] Russell R (2020). Loss of the transcription factor MAFB limits β-cell derivation from human PSCs. Nat Commun.

[9] Suissa Y (2013). Gastrin: a distinct fate of neurogenin3 positive progenitor cells in the embryonic pancreas. PLoS One.

[10] Hunter CS, Stein RW (2017). Evidence for loss in identity, de-differentiation, and trans-differentiation of islet β-cells in type 2 diabetes. Front Genet.

[11] Kaestner KH (2021). What is a beta cell. Mol Metab.

[12] Swisa A (2017). Metabolic stress and compromised identity of pancreatic beta cells. Front Genet.

[13] White MG (2013). Expression of mesenchymal and α-cell phenotypic markers in islet β-cells in recently diagnosed diabetes. Diabetes Care.

[14] Yoneda S (2013). Predominance of β-cell neogenesis rather than replication in humans with an impaired glucose tolerance and newly diagnosed diabetes. J Clin Endocrinol Metab.

[15] Harmon JS (2005). Oxidative stress-mediated, post-translational loss of MafA protein as a contributing mechanism to loss of insulin gene expression in glucotoxic beta cells. J Biol Chem.

[16] Thorens B (2015). Ins1(Cre) knock-in mice for beta cell-specific gene recombination. Diabetologia.

[17] Magnuson MA, Osipovich AB (2013). Pancreas-specific Cre driver lines and considerations for their prudent use. Cell Metab.

[18] Zhang C (2005). MafA is a key regulator of glucose-stimulated insulin secretion. Mol Cell Biol.

[19] Luan C (2019). The calcium channel subunit gamma-4 is regulated by MafA and necessary for pancreatic beta-cell specification. Commun Biol.

[20] Kim-Muller JY (2016). Aldehyde dehydrogenase 1a3 defines a subset of failing pancreatic β cells in diabetic mice. Nat Commun.

[21] Accili D (2016). When β-cells fail: lessons from dedifferentiation. Diabetes Obes Metab.

[22] Kuo T (2019). Induction of α cell-restricted Gc in dedifferentiating β cells contributes to stress-induced β-cell dysfunction. JCI Insight.

[23] Shimizu K (1999). Expression of cholecystokinin in the pancreas during development. Pancreas.

[24] Roth Flach RJ (2016). Protein kinase mitogen-activated protein kinase kinase kinase kinase 4 (MAP4K4) promotes obesity-induced hyperinsulinemia. J Biol Chem.

[25] Swisa A (2017). PAX6 maintains β cell identity by repressing genes of alternative islet cell types. J Clin Invest.

[26] Shirakawa J (2022). E2F1 transcription factor mediates a link between fat and islets to promote β cell proliferation in response to acute insulin resistance. Cell Rep.

[27] Tschop M, Heiman ML (2001). Rodent obesity models. Exp Clin Endocrinol Diabetes.

[28] Chan JY (2013). Failure of the adaptive unfolded protein response in islets of obese mice is linked with abnormalities in β-cell gene expression and progression to diabetes. Diabetes.

[29] Ravassard P (2011). A genetically engineered human pancreatic β cell line exhibiting glucose-inducible insulin secretion. J Clin Invest.

[30] Scharfmann R (2014). Development of a conditionally immortalized human pancreatic β cell line. J Clin Invest.

[31] Dahan T (2017). Pancreatic β-cells express the fetal islet hormone gastrin in rodent and human diabetes. Diabetes.

[32] Zhang P (2019). Dissecting the single-cell transcriptome network underlying gastric premalignant lesions and early gastric cancer. Cell Rep.

[33] Pullen TJ (2017). Analysis of purified pancreatic islet beta and alpha cell transcriptomes reveals 11β-hydroxysteroid dehydrogenase (Hsd11b1) as a novel disallowed gene. Front Genet.

[34] Dai C (2016). Stress-impaired transcription factor expression and insulin secretion in transplanted human islets. J Clin Invest.

[35] Cataldo LR (2022). MAFA and MAFB regulate exocytosis-related genes in human β-cells. Acta Physiol (Oxf).

[36] Pasquali L (2014). Pancreatic islet enhancer clusters enriched in type 2 diabetes risk-associated variants. Nat Genet.

[37] Mularoni L (2017). The pancreatic islet regulome browser. Front Genet.

[38] Boushey RP (2003). Hypoglycemia, defective islet glucagon secretion, but normal islet mass in mice with a disruption of the gastrin gene. Gastroenterology.

[39] Wang TC (1993). Pancreatic gastrin stimulates islet differentiation of transforming growth factor alpha-induced ductular precursor cells. J Clin Invest.

[40] Zhang M (2016). Growth factors and medium hyperglycemia induce Sox9+ ductal cell differentiation into β cells in mice with reversal of diabetes. Proc Natl Acad Sci U S A.

[41] Gaudreau MC (2022). Gastrin producing syngeneic mesenchymal stem cells protect non-obese diabetic mice from type 1 diabetes. Autoimmunity.

[42] Dalboge LS (2014). The novel GLP-1-gastrin dual agonist ZP3022 improves glucose homeostasis and increases β-cell mass without affecting islet number in db/db mice. J Pharmacol Exp Ther.

[43] Skarbaliene J (2017). In-vitro and in-vivo studies supporting the therapeutic potential of ZP3022 in diabetes. Eur J Pharmacol.

[44] Lenz A (2019). Islets from human donors with higher but not lower hemoglobin A1c levels respond to gastrin treatment in vitro. PLoS One.

[45] Matsuoka TA (2010). Regulation of MafA expression in pancreatic beta-cells in db/db mice with diabetes. Diabetes.

[46] Gupta D (2017). Temporal characterization of β cell-adaptive and -maladaptive mechanisms during chronic high-fat feeding in C57BL/6NTac mice. J Biol Chem.

[47] Winn NC (2022). Weight cycling impairs pancreatic insulin secretion but does not perturb whole-body insulin action in mice with diet-induced obesity. Diabetes.

[48] Robertson R (2007). Chronic oxidative stress as a mechanism for glucose toxicity of the beta cell in type 2 diabetes. Cell Biochem Biophys.

[49] Oetjen E (2007). Inhibition of MafA transcriptional activity and human insulin gene transcription by interleukin-1beta and mitogen-activated protein kinase kinase kinase in pancreatic islet beta cells. Diabetologia.

[50] Guo S (2013). Inactivation of specific β cell transcription factors in type 2 diabetes. J Clin Invest.

[51] Bonnefond A (2023). Monogenic diabetes. Nat Rev Dis Primers.

[52] Zhang H (2021). Monogenic diabetes: a gateway to precision medicine in diabetes. J Clin Invest.

[53] Gao T (2014). Pdx1 maintains β cell identity and function by repressing an α cell program. Cell Metab.

[54] Taylor BL (2013). Nkx6.1 is essential for maintaining the functional state of pancreatic beta cells. Cell Rep.

[55] Gutierrez GD (2017). Pancreatic β cell identity requires continual repression of non-β cell programs. J Clin Invest.

[56] Jennings RE (2020). Transcription factors that shape the mammalian pancreas. Diabetologia.

[57] Gannon M (2018). Sex differences underlying pancreatic islet biology and its dysfunction. Mol Metab.

[58] Saber N (2018). Sex differences in maturation of human embryonic stem cell-derived β cells in mice. Endocrinology.

[59] Brownrigg GP (2023). Sex differences in islet stress responses support female β cell resilience. Mol Metab.

[60] Walker EM (2021). Sex-biased islet β cell dysfunction is caused by the MODY MAFA S64F variant by inducing premature aging and senescence in males. Cell Rep.

[61] Stancill JS (2019). Transgene-associated human growth hormone expression in pancreatic β-cells impairs identification of sex-based gene expression differences. Am J Physiol Endocrinol Metab.

[62] Nomura S (2005). Alterations in gastric mucosal lineages induced by acute oxyntic atrophy in wild-type and gastrin-deficient mice. Am J Physiol Gastrointest Liver Physiol.

[63] Choi E (2015). Dynamic expansion of gastric mucosal doublecortin-like kinase 1-expressing cells in response to parietal cell loss is regulated by gastrin. Am J Pathol.

[64] Benner C (2014). The transcriptional landscape of mouse beta cells compared to human beta cells reveals notable species differences in long non-coding RNA and protein-coding gene expression. BMC Genomics.

[65] Hao Y (2021). Integrated analysis of multimodal single-cell data. Cell.

[66] Paldor M (2022). Single-cell transcriptomics reveals a senescence-associated IL-6/CCR6 axis driving radiodermatitis. EMBO Mol Med.

[67] Baron M (2016). A single-cell transcriptomic map of the human and mouse pancreas reveals inter- and intra-cell population structure. Cell Syst.

[68] Xin Y (2016). Use of the Fluidigm C1 platform for RNA sequencing of single mouse pancreatic islet cells. Proc Natl Acad Sci U S A.

[69] Tabula Muris C (2018). Single-cell transcriptomics of 20 mouse organs creates a Tabula Muris. Nature.

[70] Mawla AM, Huising MO (2019). Navigating the depths and avoiding the shallows of pancreatic islet cell transcriptomes. Diabetes.

[71] Dickerson MT (2022). G_i/o_ protein-coupled receptor inhibition of beta-cell electrical excitability and insulin secretion depends on Na^+^/K^+^ ATPase activation. Nat Commun.

[72] Walker J (2020). Integrated human pseudoislet system and microfluidic platform demonstrate differences in GPCR signaling in islet cells. JCI Insight.

