# Supplementary material for: Species-specific roles for the MAFA and MAFB transcription factors in regulating islet β cell identity
Source: JCI Insight. 2023 Aug 22;8(16):e166386. doi: 10.1172/jci.insight.166386 (PMC10543725; doi:10.1172/jci.insight.166386)
Supplement: Supplemental data [file jciinsight-8-166386-s139.pdf]

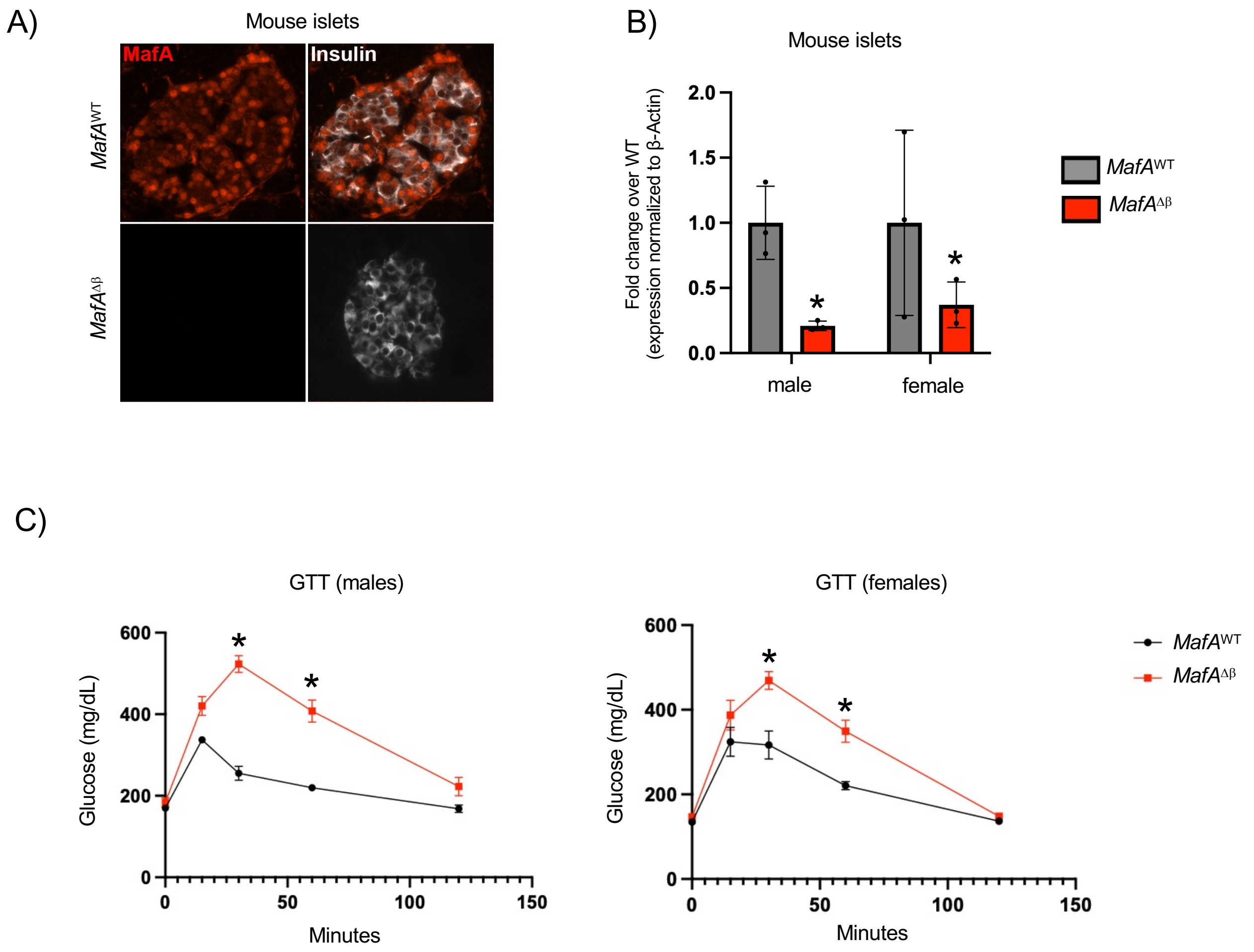

Supplemental Figure 1

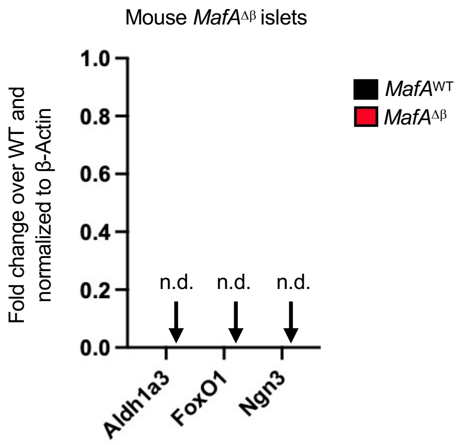

Supplemental Figure 2

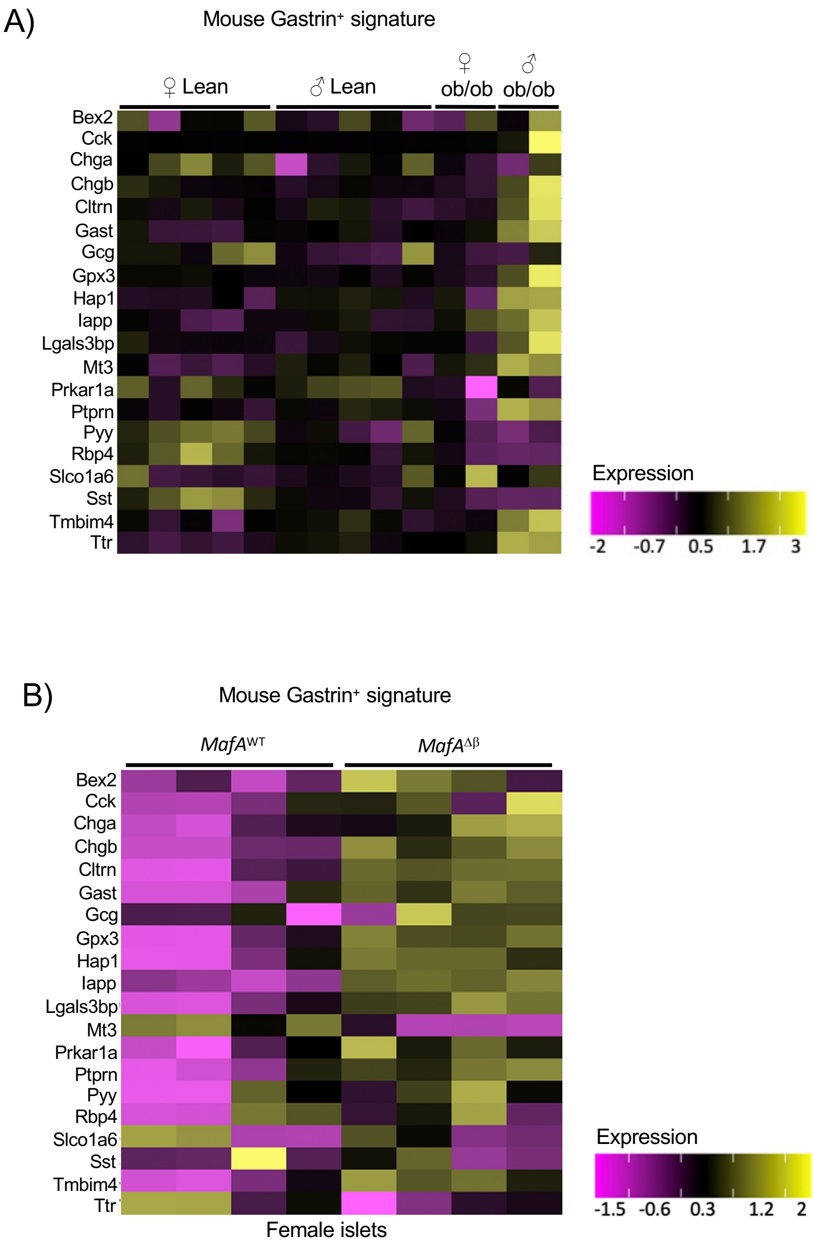

Supplemental Figure 3

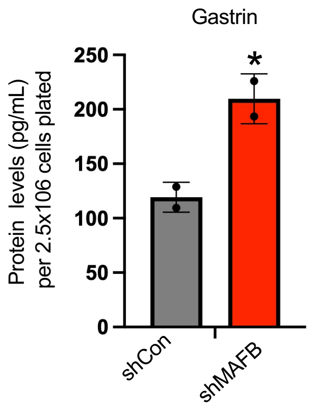

Supplemental Figure 4

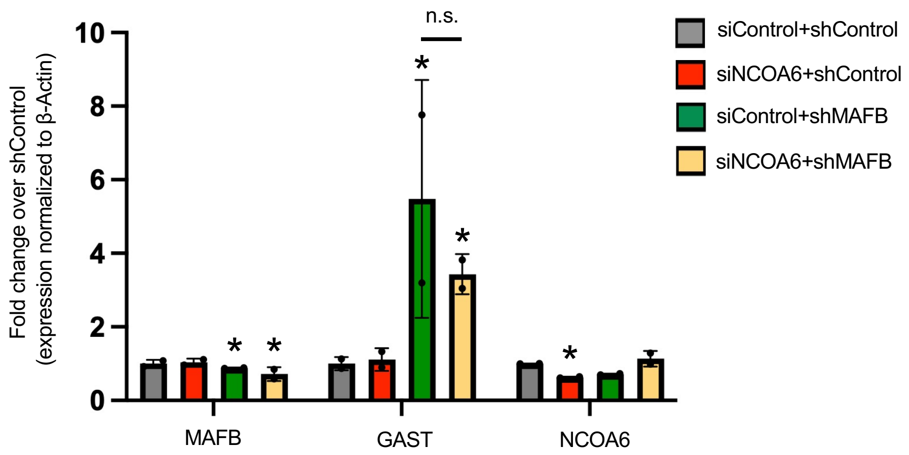

Supplemental Figure 5

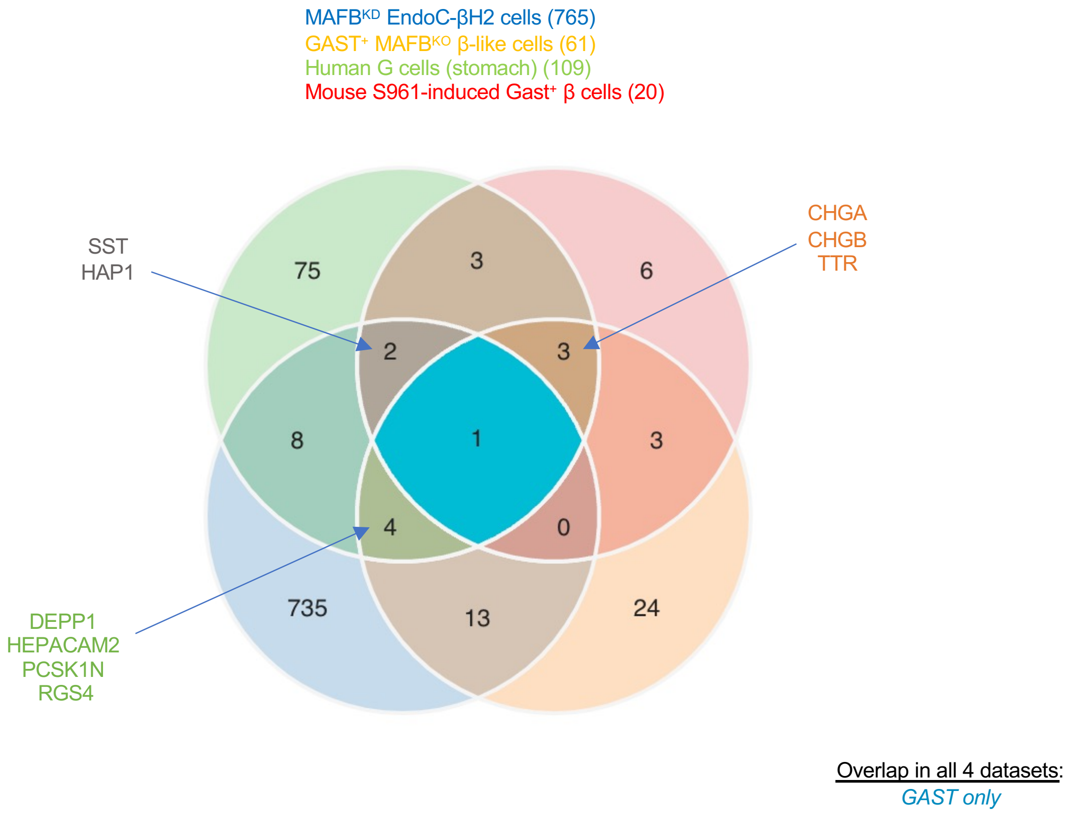

Supplemental Figure 6

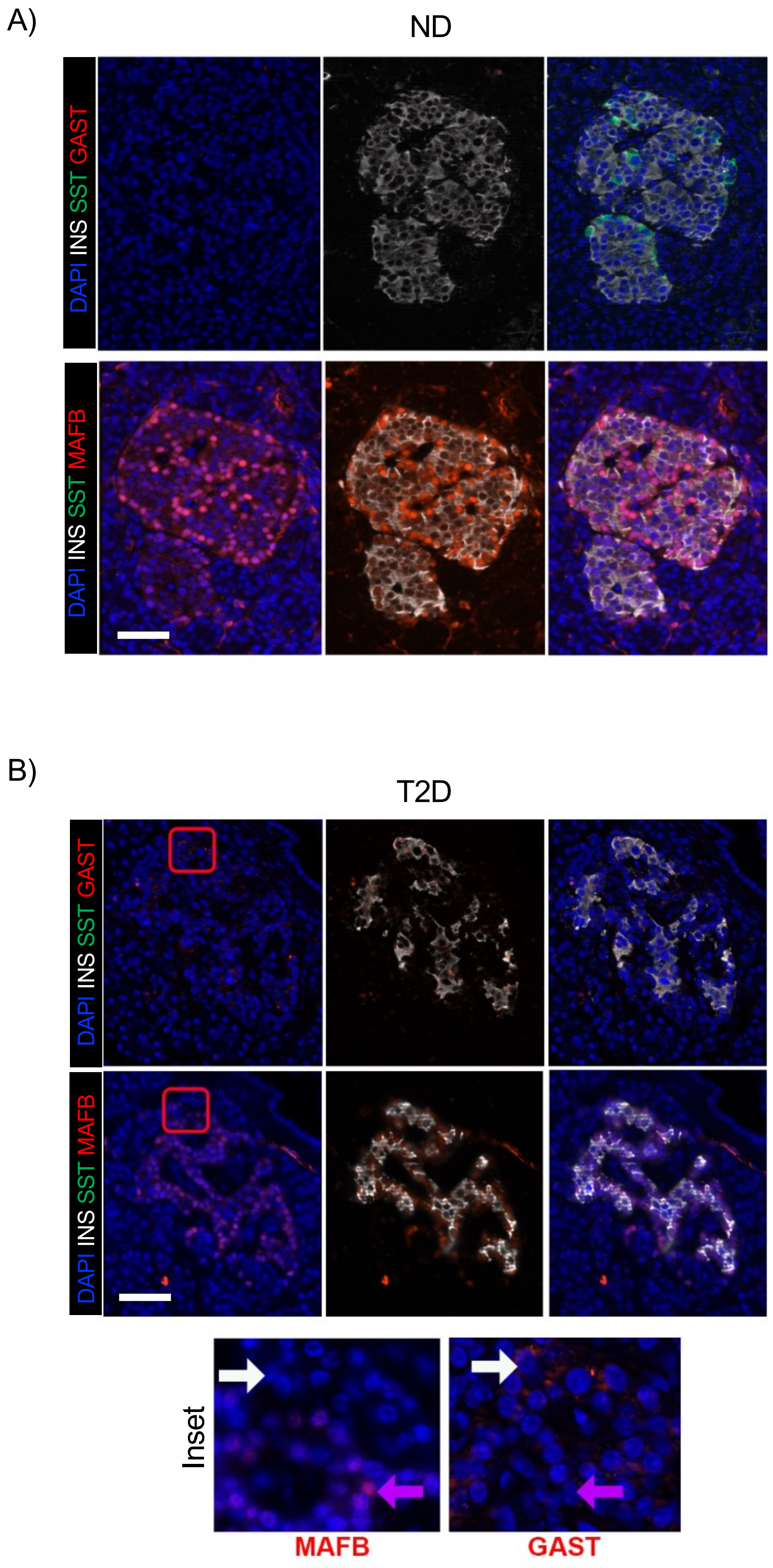

Supplemental Figure 7

A)

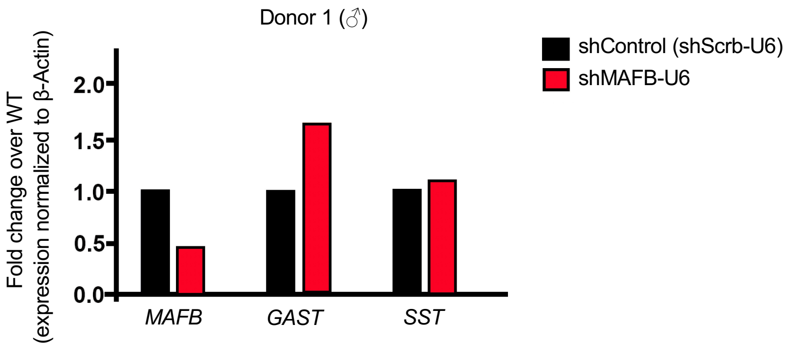

B)

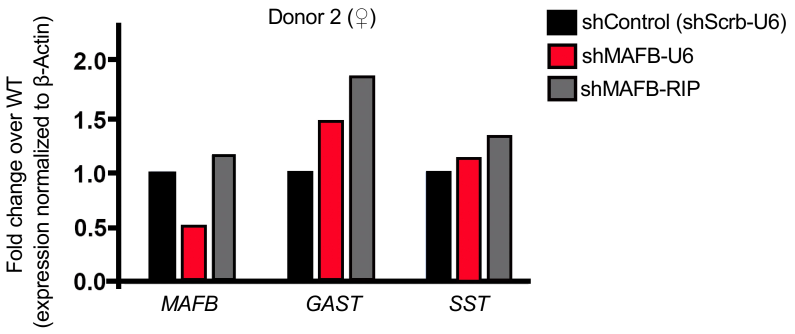

Supplemental Figure 8

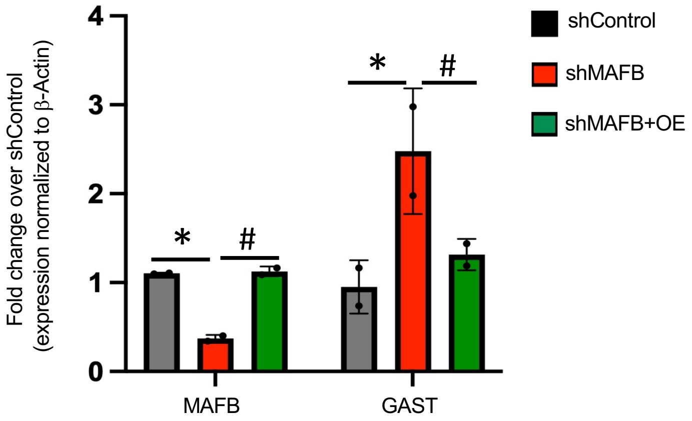

Supplemental Figure 9

Supplemental Figure 1: *MafA*$^{\Delta\beta}$ mice are glucose intolerant

(A) Immunostaining for MafA (red) and Ins (white) in 3-month-old male WT and *MafA*$^{\Delta\beta}$ pancreata. n=3 animals per group, and experiment repeated twice. Bar, 50μm.

(B) *MafA* mRNA levels in WT and *MafA*$^{\Delta\beta}$ mouse islets. n=3-4 animals per group, and experiment repeated twice. Mean ± SEM. *p<0.05 by Student's t-test.

(C) IPGTT analysis of WT and *MafA*$^{\Delta\beta}$ male and female mice. n=3-4 animals per group. Mean ± SEM at each time point. *p<0.05 by Student's t-test at the same time points.

Supplemental Figure 2: Aldh1a3, Foxo1, and Ngn3 are undetectable in *MafA*$^{\Delta\beta}$ islets. qPCR analysis of Aldh1a3, Foxo1, and Ngn3 de-differentiation markers in *MafA*$^{\Delta\beta}$ mouse islets. n=3-4 animals per group and sex. Mean ± SEM. n.d., not detected.

Supplemental Figure 3: The S961-induced Gast^{+} gene signature is maintained in male, but not female, *ob/ob* mouse islets.

(A) The presence of elevated Gast^{+} signature genes were determined by analysis of the RNA-seq datasets prepared from FACS-sorted β cells of lean female (n=5 animals), lean male (n=5 animals), *ob/ob* female mice (n=2 animals), and *ob/ob* male mice (n=2 animals). The islet Gast^{+} gene signature from S961-treated mice (**Supplemental Table 1**) largely overlapped with those in male and not female *ob/ob* mouse islet β cells.

(B) Heatmap of Gast^{+} signature genes in female *MafA*$^{\Delta\beta}$ islets. n=4 animals/condition.

Supplemental Figure 4: GAST protein production in MAFB^{KD} EndoC-βH2 cells.

GAST protein was produced at higher levels in MAFB^{KD} EndoC-βH2 cells than shControl-treated cells. Human GAST was analyzed by ELISA in this representative graph. Mean ± SEM. n=2 replicates/group, and ELISA was repeated twice. *p<0.05 by Student's t-test.

Supplemental Figure 5: The MLL3/4 coregulator did not influence MAFB-mediated repression of *GAST* expression.

Representative qPCR analysis of EndoC-βH2 cells subject to MAFB and/or NCOA6 knockdown by shRNA and siRNA, respectively. Reduction of the core MLL3/4 subunit, NCOA6, did not accentuate *GAST* upregulation by MAFB reduction. Mean ± SEM. n=2 replicates/group, and experiment was repeated 3 times. *$p<0.05$ by Student's t-test, compared to siControl+shControl group. n.s., not significant.

Supplemental Figure 6: Venn diagram analysis of the DEGs expressed in Gast^{+} cells of S961-treated mouse islets, human MAFB^{KO} β-like cells, and human stomach G cells with the broader MAFB^{KD} EndoC-βH2 cell population. Only Gast itself was enriched in all four datasets.

Supplemental Figure 7: GAST is produced in female MAFB-deficient T2D islet cells.

(A-B) Representative image of immunostaining performed on serial sections to detect GAST (Red in top panels), MAFB (Red in bottom panels), SST (Green), INS (White), and nuclei (blue). GAST^{+} cells were not detected in female healthy donor islets but were in T2D islets. Insets show magnified view of rare GAST^{+}MAFB^{-} (White arrows) and GAST^{-}MAFB^{+} (Purple arrows) cells. Bar, 100μm.

Supplemental Figure 8: *GAST* is increased in MAFB^{KD} human pseudoislets.

(A-B) qPCR analysis of whole pseudoislets (n=2 donors) showed increased *GAST* expression upon targeting MAFB either in all islet cells (shMAFB-U6) or only β cells (shMAFB-RIP) in relation to the scramble control (shControl). Donor information in **Supplemental Table 3**.

Supplemental Figure 9: Overexpression of MAFB in $MAFB^{KD}$ EndoC-βH2 cells masks GAST induction. Representative qPCR analysis of EndoC-βH2 cells subject to MAFB knockdown with or without MAFB overexpression (OE). Mean ± SEM. n=2 replicates/group, and experiment was repeated twice. *$p<0.05$ by Student's t-test compared to shControl. $^{\#}p<0.05$ by Student's t-test compared to shMAFB group.

Supplemental Table 1: Mouse $Gast^{+}$ β cell signature (male) table

Supplemental Table 2: Human $GAST^{+}$ β cell signature (male) table

Supplemental Table 3: Human islet donor characteristics table

Supplemental Table 4: qPCR primer table

Supplemental Table 5: Primary antibody table

Supplemental Table 6: Competitor probe sequences table

Supplemental Table 7: Gene markers used to identify unique cell clusters in hESC-derived $MAFA^{WT}$ β-like cells

Supplemental Table 8: Gene markers used to identify unique cell clusters in hESC-derived $MAFA^{KO}$ β-like cells
